# Effects of exposure to surrounding green, air pollution and traffic noise with non-accidental and cause-specific mortality in the Dutch national cohort

**DOI:** 10.1186/s12940-021-00769-0

**Published:** 2021-07-14

**Authors:** Jochem O. Klompmaker, Nicole A. H. Janssen, Lizan D. Bloemsma, Marten Marra, Erik Lebret, Ulrike Gehring, Gerard Hoek

**Affiliations:** 1 National Institute for Public Health and the Environment (RIVM), Antonie van Leeuwenhoeklaan 9, 3721 MA Bilthoven, The Netherlands; 2 Institute for Risk Assessment Sciences (IRAS), Utrecht University, Utrecht, The Netherlands

**Keywords:** Greenness, Air pollution, Noise, Combined effects, Mortality

## Abstract

**Background:**

Everyday people are exposed to multiple environmental factors, such as surrounding green, air pollution and traffic noise. These exposures are generally spatially correlated. Hence, when estimating associations of surrounding green, air pollution or traffic noise with health outcomes, the other exposures should be taken into account. The aim of this study was to evaluate associations of long-term residential exposure to surrounding green, air pollution and traffic noise with mortality.

**Methods:**

We followed approximately 10.5 million adults (aged ≥ 30 years) living in the Netherlands from 1 January 2013 until 31 December 2018. We used Cox proportional hazard models to evaluate associations of residential surrounding green (including the average Normalized Difference Vegetation Index (NDVI) in buffers of 300 and 1000 m), annual average ambient air pollutant concentrations [including particulate matter (PM_2.5_), nitrogen dioxide (NO_2_)] and traffic noise with non-accidental and cause-specific mortality, adjusting for potential confounders.

**Results:**

In single-exposure models, surrounding green was negatively associated with all mortality outcomes, while air pollution was positively associated with all outcomes. In two-exposure models, associations of surrounding green and air pollution attenuated but remained. For respiratory mortality, in a two-exposure model with NO_2_ and NDVI 300 m, the HR of NO_2_ was 1.040 (95%CI: 1.022, 1.059) per IQR increase (8.3 µg/m^3^) and the HR of NDVI 300 m was 0.964 (95%CI: 0.952, 0.976) per IQR increase (0.14). Road-traffic noise was positively associated with lung cancer mortality only, also after adjustment for air pollution or surrounding green.

**Conclusions:**

Lower surrounding green and higher air pollution were associated with a higher risk of non-accidental and cause-specific mortality. Studies including only one of these correlated exposures may overestimate the associations with mortality of that exposure.

**Supplementary Information:**

The online version contains supplementary material available at 10.1186/s12940-021-00769-0.

## Introduction

Everyday people are exposed to multiple environmental factors that can affect their health. Repeated and prolonged exposures can contribute to pathophysiological changes, thereby influencing the development of diseases over the life course of individuals, eventually leading to premature death [1]. The sum of all exposures to which an individual is subjected from conception to death is called the exposome [2]. The exposome consists of different domains, including (but not restricted to) environmental exposures, such as air pollution, traffic noise and surrounding green. Air pollution has been associated with increased non-accidental and cause-specific mortality, such as cardiovascular and respiratory disease mortality [3–5]. Some studies have also found associations of air pollution with increased incidence of dementia, Parkinson’s and Alzheimer’s disease [6–9]. Traffic noise has also been linked to increased non-accidental and cardiovascular disease mortality [10–13]. On the other hand, surrounding green has been associated with decreased non-accidental and cause-specific mortality [14–17].

Exposures to surrounding green, air pollution and traffic noise are generally spatially correlated [18]. Air pollution and road-traffic noise share motorized traffic as a common source and are therefore positively correlated. The absence of air pollution and traffic noise sources in green areas and the limitation of transmission of emissions generally result in negative correlations between air pollution, traffic noise and surrounding green. Hence, information about the risk of one of these exposures could be partly attributed to another correlated exposure, and estimating associations of surrounding green, air pollution and traffic noise using single-exposure models can result in an overestimation of effects [19–21].

Most studies evaluating mortality effects focus only on either surrounding green, air pollution or traffic noise [10, 12–17, 22–24]. To control for potential confounding by the other, spatially correlated environmental exposures, adjustments for these other exposures are generally made. However, most studies do not report associations of the other environmental exposures with mortality and therefore the mutual impact of exposures remains unknown. We previously evaluated associations of surrounding green, air pollution and traffic noise with non-accidental and cause-specific mortality in a Dutch population-based survey of more than 300,000 participants over a five year follow-up period and found no associations [18]. We speculated that potential selection bias and the overrepresentation of the elderly in combination with the relatively short follow-up period may have resulted in the lack of relationships in this population-based survey [18]. To overcome these limitations, we linked long-term surrounding green, air pollution and traffic noise exposure to a Dutch national cohort, which includes the full Dutch population aged ≥ 30 years on January 1, 2013 (~ 10.5 million). In previous studies in a Dutch national cohort, associations between air pollution and mortality have been reported, but surrounding green and traffic noise were not considered in these studies [22–24]. The aim of this study was to evaluate associations of long-term residential exposure to surrounding green, air pollution and traffic noise with non-accidental and cause-specific mortality.

## Methods

### Study population and outcome definition

We created an administrative cohort that includes the full Dutch population aged 30 + , on January 1, 2013. The cohort was compiled based on data from several databases from Statistics Netherlands (CBS), including the longitudinal mortality registry for follow-up, individual covariates (such as sex, marital status, region of origin and standardized household income) and regional socio-economic status (SES) covariates. To adjust for potential confounders not accounted for by individual SES indicators, we linked several neighborhood (n ~ 2600, representing on average approximately 2900 addresses) and regional-level (NUTS 3, *n* = 40) SES indicators to the cohort, similar to our previous studies [18, 24]. NUTS (Nomenclature des Unités Territoriales Statistique) is a geocode standard for referencing the subdivisions of countries for statistical purposes and is developed and regulated by Eurostat, the statistical office of the European Union. There are 40 NUTS3 regions in the Netherlands. We linked mean income (mean income per income recipient), percentage of (non-western) immigrants and unemployment rate (number of people with income support per 1000 inhabitants aged 15–64 years) at regional and neighborhood level to our cohort. Further, we used a composite SES score that represents the educational, occupational and economical status at a four digit postal code level (PC4, n ~ 4000, representing on average approximately 1800 addresses). This composite score was only available at PC4 level; hence we do not have the composite score at regional or neighborhood level. The follow-up period was from 1 January 2013 until 31 December 2018.

We selected non-accidental mortality [International Classification of Diseases, 10th Revision (ICD-10) codes: A00-R99], circulatory disease mortality (I00-I99), respiratory disease mortality (J00-J99), lung cancer mortality (C34) and neurodegenerative disease mortality [including dementia (F00-F03), motor neuron disease (G12.2), Parkinson’s disease (G20-G22), Alzheimer’s disease (G30), Multiple sclerosis (G35)] as our main outcomes. Secondary analyses were conducted with more specific mortality outcomes: ischemic heart disease mortality (I20-I25; IHD), cerebrovascular mortality (I60-I69), COPD mortality (J40-J44) and dementia mortality (F00-F03)]. All mortality outcomes were identical to the mortality outcomes we used in our previously published study [18].

From 2013, Statistics Netherlands (CBS) switched from manual coding of mortality statistics to an automatic system (IRIS) for the selection of the underlying cause of death [25]. IRIS automatically selects the underlying cause of death when multiple causes of death are reported based on internationally agreed decision tables. Differences in manual and automatic coding is the rationale for starting the follow-up of the cohort in 2013.

### Exposure assessment

#### Surrounding green

Residential surrounding green was assessed by two different green metrics. The Normalized Difference Vegetation Index (NDVI) was used to assess surrounding greenness, i.e., the average density of green vegetation within a circular buffer of each residential address. The NDVI was derived from Landsat 5 images from the summer of 2010 and captures the density of green vegetation at a spatial resolution of 30 m. NDVI values range between -1 and 1, with higher numbers indicating a higher density of green vegetation. Negative NDVI values represent water and were set to zero [26]. We combined cloud free images from the summer of 2010 to create a map that covers (almost) the whole country. Additionally, a highly detailed national land-use database of the Netherlands (TOP10NL) of 2010 [27] was used to assess surrounding green space, i.e., the proportion of green space within a buffer around the participant’s residential address. TOP10NL is developed by the Netherlands’ Cadastre, Land Registry and Mapping Agency (Kadaster) and is based on high resolution aerial photos, external databases and field surveying. TOP10NL divides the Netherlands into polygons with different classes of land-use (water, road and terrain). The terrain class is divided in 21 subclasses; 11 of these classes correspond to green areas (cropland, orchard, plant nursery, mixed forest, willow forest, deciduous forest, coniferous forest, fruit farm, grassland, heather and poplar). We have aggregated these 11 classes into one overall green class. TOP10NL does, in contrast to NDVI, not include private green property (such as gardens) and street greenery. Average surrounding greenness and surrounding green space were assessed in buffers with a 300 and 1000 m radius, following our previous work [26].

#### Air pollution

For each address in the Netherlands, long-term average ambient concentrations of several air pollutants were predicted by three different models [24]. Particulate matter with aerodynamic diameter ≤ 2.5 µm (PM_2.5_) and nitrogen dioxide (NO_2_) concentrations were estimated with national land-use regression (LUR) [28, 29], Europe-wide hybrid [30] and dispersion [31–33] models. LUR and Europe-wide hybrid models also estimated black carbon (BC, measured as PM_2.5_ absorbance) concentrations, while dispersion models estimated elemental carbon concentrations (µg/m^3^). We converted elemental carbon concentrations to BC concentrations using the average conversion factor reported Janssen et al. (2011) (1 unit BC = 1.1 µg/m^3^ elemental carbon) [34]. NO_2_ concentrations estimated by the LUR model higher than 80 µg/m^3^ (n ~ 500) were set to 80 µg/m^3^ as these values are probably due to an unrealistic combination of explanatory variables (the maximum annual average NO_2_ concentration measured within the ESCAPE study is 61.5 ug/m^3^). As we have no evidence which model predicts residential concentrations of PM_2.5_, NO_2_ and BC best [24], we decided to average the residential concentration of PM_2.5_, BC and NO_2_ of the three models. Long-term average concentrations of PM_10_, PM_coarse_ and two Oxidative Potential (OP) metrics, electron spin resonance (OP^ESR^) and dithiothreitol (OP^DTT^), were estimated by national LUR models [29, 35]. OP is an intrinsic measure of PM to oxidize target molecules and thus effectively incorporates biologically relevant properties of PM [35].

#### Traffic noise

Residential traffic noise levels were assessed by the Standard Model Instrumentation for Noise Assessments (STAMINA). STAMINA is a model to map environmental noise in the Netherlands. This model was developed at the Dutch National Institute for Public Health and the Environment (RIVM) and uses the standard Dutch Calculation method for traffic and industrial noise [36]. The spatial resolution of the noise maps depends on the distance between source and observation point. The lowest resolution is 80 × 80 m, and close to the source the level of detail is highest, with a resolution of 10 × 10 m [36]. Daily average (24 h, Lden) and night-time average (23:00—07:00 h, Lnight) road- and rail-traffic noise exposures were assessed for 2011. Since correlations between Lden and Lnight were high (spearman rho = 0.99 for road-traffic and 0.95 for rail-traffic noise), we only used Lden in our analyses.

### Statistical analyses

Since we did not have land-use data across the border of the Netherlands, subjects with residential addresses within 1 km (largest buffer) of the border of the Netherlands or outside the NDVI map were excluded from our cohort (~ 1.7%). Furthermore, we excluded subjects with missing exposure data, resulting in a study population of 10,481,566 subjects.

To study whether surrounding green, air pollution and traffic noise were associated with mortality, we used Cox proportional hazard models. We specified a priori Cox models with age as underlying time scale, stratified by sex and adjusted for marital status, region of origin, standardized household income, composite SES at a four digit postal code level (PC4), mean income per income recipient of the neighborhood and the region, unemployment rate of the neighborhood and the region and percentage of immigrants of the neighborhood and the region [24]. Categories of covariates in the Cox models were identical to categories presented in Table 1, except for the area-level SES covariates (quintiles). We evaluated the shape of the exposure–response curves by using natural splines with 3 degrees of freedom. For most exposure–response curves (Figure S1, Additional file 1), deviations from linearity were only found in the extremes of the distribution with sparse data. We present the linear effect of exposure to surrounding green, air pollution and traffic noise per interquartile range (IQR) to allow comparison of effect sizes across exposures.

**Table 1:** Population characteristics (*n* = 10,481,566)

Covariate	Category	N (proportion) or median (IQR)
**Individual covariates**		
Age		53 (23)
Sex	male	5,109,777 (0.49)
	female	5,371,789 (0.51)
Marital status	married	6,385,590 (0.61)
	widowed	821,282 (0.08)
	divorced	1,147,403 (0.11)
	single	2,127,291 (0.20)
Region of origin	Morocco	163,553 (0.02)
	Turkey	197,937 (0.02)
	Suriname	199,253 (0.02)
	Antilles Netherlands	67,021 (0.01)
	Other non-western	325,276 (0.03)
	western	1,026,870 (0.10)
	Netherlands	8,501,656 (0.81)
Standardized household income	< 1%	53,471 (0.01)
	1–5%	135,758 (0.01)
	5–10%	335,972 (0.03)
	10–25%	1,280,658 (0.12)
	25–50%	2,555,622 (0.25)
	50–75%	2,810,407 (0.27)
	75–90%	1,813,078 (0.18)
	90–95%	633,437 (0.06)
	95–99%	517,220 (0.05)
	> 99%	128,445 (0.01)
**Area-level SES covariates**		
Composite SES 4 digit postal code	Based on education, income and paid occupation (year = 2011–2014)	0.3 (1.3)
Mean income neighborhood	Mean income per income recipient *€ 1000 (year = 2010)	29.3 (5.4)
Unemployment rate neighborhood	Number of people with income support per 1000 inhabitants of 15–64 years (year = 2010)	25.0 (10.0)
Percentage non-western immigrants neighborhood	Percentage non-western immigrants (year = 2010)	7.0 (11.0)
Mean income region	Mean income per income recipient *€ 1000 (year = 2010)	34.1 (2.6)
Unemployment rate region	Number of people with income support per 1000 inhabitants of 15–64 years (year = 2010)	24.5 (7.3)
Percentage non-western immigrants region	Percentage non-western immigrants (year = 2010)	8.4 (5.9)
**Mortality outcomes**		
Non-accidental mortality		776,021 (0.07)
Circulatory disease mortality		215,018 (0.02)
Ischemic heart disease mortality		49,530 (0.00)
Cerebrovascular disease mortality		52,276 (0.00)
Respiratory disease mortality		69,401 (0.01)
COPD mortality		37,203 (0.00)
Lung cancer mortality		59,837 (0.01)
Neurodegenerative disease mortality		90,587 (0.01)
Dementia mortality		57,122 (0.01)
**Exposures**		
NDVI 300 m (unitless)		0.52 (0.14)
TOP10NL 300 m (proportion)		0.18 (0.23)
NDVI 1000 m (unitless)		0.55 (0.14)
TOP10NL 1000 m (proportion)		0.34 (0.31)
NO_2_ (µg/m^3^) ^a^		26.3 (8.3)
PM_2.5_ (µg/m^3^) ^a^		16.8 (1.4)
BC (10^–5^/m) ^a^		1.3 (0.3)
PM_10_ (µg/m^3^) ^b^		24.5 (1.3)
PM_coarse_ (µg/m^3^) ^b^		8.1 (0.8)
OP^DTT^ (nmol DTT/min/m^3^) ^b^		1.2 (0.3)
OP^ESR^ (A.U./1000/m^3^) ^b, c^		0.9 (0.2)
Road traffic noise (L_den_, dB)		53.5 (7.5)
Rail-traffic noise (L_den_, dB)		30.7 (9.4)

^a ^Estimated with the average of the national LUR, Europe-wide hybrid and national dispersion models. NO2 = nitrogen dioxide, PM2.5 = particulate matter with aerodynamic diameter ≤ 2.5 µm, BC = Black carbon

^b ^Estimated with a national LUR model, PM10 = particulate matter with aerodynamic diameter ≤ 10 µm, PMcoarse = particulate matter with aerodynamic diameter between 10 and 2.5 µm, OP^ESR^ = Oxidative Potential electron spin resonance, OP^DTT^ = Oxidative Potential dithiothreitol

^c ^A.U. = arbitrary unit

#### Single-exposure models

To evaluate the impact of potential confounders, we specified Cox models with increasing degrees of covariate adjustment. Model 1 included the baseline hazard stratified by sex, Model 2 was additionally adjusted for the other individual level covariates (marital status, country of origin and standardized household income). Composite SES score at PC4 level was included in Model 3. Model 4 (main model) additionally included the other area-level SES covariates (mean income per income recipient of the region and of the neighborhood, unemployment rate of the region and of the neighborhood and percentage non-western immigrants of the region and of the neighborhood). Model 3 was specified for consistency with an earlier analysis in a Dutch national cohort [22, 23].

As sensitivity analyses, we included a correction of the standard errors for clustering of individuals in neighborhoods [24], we additionally adjusted for degree of urbanization [non-urban (< 1500 addresses/km^2^) vs. urban (≥ 1500 addresses/km^2^)] and for indicators of geographical NUTS 1 regions of the Netherlands (North, East, West, and South) to adjust for potential regional patterns in mortality not accounted for by our covariates. We also ran models for a subset of our study population including only individuals who did not move in the 5 years preceding the start of the follow-up period (*n* = 7,778,896; ~ 75% of the total population).

As we did not have information on smoking status and BMI, we used an indirect adjustment technique developed by Shin et al. to indirectly adjust for smoking status and body mass index (BMI) [37]. The method uses information contained within a representative ancillary dataset regarding the multivariate relationships between the missing lifestyle covariates (dependent variable) and the (air pollution, surrounding green and noise) exposure, adjusting for observed covariates in our main model 4 [37, 38]. We used a randomly stratified sample of the Public Health monitor 2012, with information about smoking status (never, ex and current smoker) and BMI (< 18.5, 18.5–24.9, 25.0–29.9, ≥ 30.0), with similar covariate distribution as our study population (Table S1, Additional file 1). Effect estimates for associations of smoking status and BMI with non-accidental mortality were obtained from a European cohort of more than 300,00 adults in the ELAPSE study [39]. As we only have information about associations of smoking status and BMI with non-accidental mortality, indirect adjustment was performed for non-accidental mortality, but not for cause-specific mortality. For further interpretation of potential bias, we specified linear models in our randomly stratified survey sample with the exposure as the dependent variable and included smoking status, BMI and all covariates included in the main model (model 4). To compare relations between exposures, we divided the beta (the difference in exposure levels between categories of smoking status and BMI) by the interquartile range (IQR) of the exposure in the full population and then multiplied by 100 to obtain percentages.

Further, we included an interaction term for age, to evaluate whether associations of environmental exposures with mortality differ between the elderly (≥ 65 years) and the non-elderly (< 65 years). Several studies showed that associations of surrounding green, air pollution or traffic noise with mortality were stronger for the non-elderly than the elderly [12, 15, 22, 40, 41].

#### Two-exposure models

The empirical correlation between surrounding green and air pollution and traffic noise is likely caused by multiple mechanisms. As the data we used in this study does not allow a clear judgement on whether relations are causal, partly causal or non-causal, we considered surrounding green, air pollution and traffic noise as mutual confounders. To evaluate potential mutual confounding of surrounding green, air pollution and traffic noise, we specified two-exposure Cox models with combinations of surrounding green, air pollution and traffic noise exposures. Three-exposure Cox models with combinations of surrounding green, air pollution and traffic noise exposures were only specified if we observed associations (in the expected direction) of all three exposures with a mortality outcome. Further, we evaluated a joint hazard ratio (JHR) using the Cumulative Risk Index (CRI) method [40, 42]. As the CRI has been developed to determine joint risks, we evaluated the effect of *decreased* surrounding green and *increased* concentrations of air pollution and levels of traffic noise. The JHR represents the hazard for a 1-unit (here IQR) *increase* in air pollution and traffic noise and a 1-unit (IQR) *decrease* for surrounding green exposure relative to the hazard for no increase (decrease for surrounding green) in any of the exposures.

We denote the JHR based on the combination of the P exposures evaluated at $x$ as the Cumulative Risk Index (CRI) and define it as:

$$CRI=exp\left\{{\sum }_{p=1}^{p}{\widehat{\beta }}_{p}{x}_{p}\right\}=\mathrm{e}\mathrm{x}\mathrm{p}\left(\widehat{\beta }\text{'}x\right)=\prod _{p=1}^{p}{JHR}_{p}$$

where ${\widehat{\beta }}^{\text{'}}=({\widehat{\beta }}_{1},\dots ,{\widehat{\beta }}_{p})$ are the estimates of the log hazard ratio for the P exposures estimated in a Cox proportional hazard model consisting of all P exposures together, ${x}^{\text{'}}=\left({x}_{1},\dots ,{x}_{p}\right)$ are the levels at which each exposure-specific HR is evaluated, ${JHR}_{p}=\mathrm{e}\mathrm{x}\mathrm{p}({\widehat{\beta }}_{p}{x}_{p})$ denotes the joint hazard ratio for the ${p}^{th}$ exposure in a two-exposure model. JHRs were estimated assuming additive effect estimates (log hazard ratios) of joint exposures. The 95% confidence interval of CRI is defined by:

$$\exp \left\{ {\hat{\beta }^{'} x \pm 1.96 \times \sqrt {x^{'} \times Cov\left( {\hat{\beta }} \right) \times x} } \right\}$$

This definition of the confidence interval is similar to that described elsewhere [40, 43].To limit the number of analyses, we decided to use NDVI 300 m, NO_2_, PM_2.5_ and road-traffic noise exposure in sensitivity and two-exposure analyses, as these are commonly studied environmental exposures. Based on results of single-exposure models, we also used TOP10NL 1000 m and OP^DTT^ in two-exposure analyses.

## Results

Our cohort consisted of 10,481,566 subjects aged ≥ 30 years who contributed 59,845,307 person-years of follow-up. We observed 776,021 non-accidental deaths (Table 1), of which approximately 28% circulatory disease deaths, 9% respiratory disease deaths and 8% lung cancer deaths. We observed 90,587 neurodegenerative disease deaths (12% of all non-accidental deaths) of which approximately 63% were dementia deaths.

The variation (IQR / median) in NDVI surrounding greenness was lower than the variation in TOP10NL surrounding green space (Table 1). Traffic-related air pollutants, such as NO_2_ and BC, varied more than PM_10_ and PM_2.5_. The variation in road-traffic noise was lower than the variation in NO_2_ and BC, but larger than the variation in PM_2.5_. Surrounding green, air pollution and traffic noise exposures were overall moderately correlated (Figure S2, Additional file 1). NDVI 300 m was negatively correlated with NO_2_ (spearman rho = -0.52), PM_2.5_ (spearman rho = -0.31) and road-traffic noise (spearman rho = -0.27).

We found negative associations of surrounding green with all main mortality outcomes (Table 2). For non-accidental mortality, we found a HR of 0.972 (95%CI: 0.969, 0.976) per IQR increase in NDVI 300 m. Associations with respiratory disease and lung cancer mortality were stronger than associations with non-accidental, circulatory and neurodegenerative disease mortality. HRs were similar for all four surrounding green measures. Air pollution was positively associated with all main mortality outcomes. For non-accidental mortality, we found a HR of 1.022 (95%CI: 1.017, 1.028) per IQR increase in NO_2_. The strongest associations were found with respiratory disease and lung cancer mortality. OP^DTT^ was the only pollutant that was positively associated with neurodegenerative disease mortality. Associations of PM_2.5_, BC and NO_2_ estimated by the individual LUR, hybrid and dispersion models with mortality were mostly similar (Table S2, Additional file 1). For road-traffic noise, we only found positive associations with non-accidental and lung cancer mortality and negative associations with neurodegenerative disease mortality. In the linear analyses, rail-traffic noise was not associated with our main outcomes, however, spline analyses showed a threshold-shape curve with neurodegenerative disease mortality (Figure S1e, Additional file 1).

**Table 2:** Associations of exposures with non-accidental and cause-specific mortality in single-exposure models ^a^

Exposure (IQR)	Non-accidental mortality	Circulatory disease mortality	Respiratory disease mortality	Lung cancer mortality	Neurodegenerative disease mortality
	**HR (95% CI)**	**HR (95% CI)**	**HR (95% CI)**	**HR (95% CI)**	**HR (95% CI)**
NDVI 300 m (0.14)	0.972 (0.969, 0.976)	0.987 (0.981, 0.994)	0.954 (0.943, 0.965)	0.926 (0.915, 0.937)	0.977 (0.967, 0.988)
TOP10NL 300 m (0.23)	0.976 (0.973, 0.979)	0.983 (0.977, 0.988)	0.962 (0.951, 0.972)	0.952 (0.942, 0.963)	0.991 (0.981, 1.000)
NDVI 1000 m (0.14)	0.977 (0.974, 0.981)	0.994 (0.987, 1.002)	0.979 (0.966, 0.992)	0.942 (0.930, 0.955)	0.982 (0.970, 0.994)
TOP10NL 1000 m (0.31)	0.966 (0.962, 0.971)	0.981 (0.973, 0.990)	0.959 (0.944, 0.974)	0.941 (0.927, 0.957)	0.984 (0.969, 0.999)
NO2 (8.3) ^b^	1.022 (1.017, 1.028)	1.013 (1.004, 1.023)	1.061 (1.043, 1.079)	1.074 (1.055, 1.092)	0.992 (0.976, 1.008)
PM2.5 (1.4) ^b^	1.013 (1.009, 1.016)	1.016 (1.009, 1.023)	1.060 (1.047, 1.073)	1.046 (1.033, 1.060)	1.009 (0.997, 1.020)
BC (0.3) ^b^	1.014 (1.010, 1.018)	1.012 (1.005, 1.020)	1.042 (1.028, 1.056)	1.051 (1.037, 1.065)	0.992 (0.980, 1.004)
PM10 (1.3) ^c^	1.004 (1.001, 1.008)	0.998 (0.992, 1.005)	1.009 (0.998, 1.021)	1.024 (1.012, 1.036)	0.984 (0.973, 0.995)
PMcoarse (0.8) ^c^	1.010 (1.006, 1.013)	1.003 (0.996, 1.009)	1.016 (1.004, 1.028)	1.024 (1.012, 1.036)	1.000 (0.988, 1.011)
OPDTT (0.3) ^c^	1.020 (1.016, 1.024)	1.013 (1.006, 1.020)	1.061 (1.048, 1.075)	1.056 (1.042, 1.069)	1.019 (1.007, 1.031)
OPESR (0.2) ^c^	1.008 (1.004, 1.011)	1.012 (1.005, 1.018)	1.028 (1.017, 1.039)	1.028 (1.017, 1.040)	0.988 (0.978, 0.999)
Road-traffic noise (7.5)	1.005 (1.002, 1.008)	1.005 (0.999, 1.011)	1.004 (0.994, 1.015)	1.032 (1.021, 1.043)	0.972 (0.962, 0.982)
Rail-traffic noise (9.4)	1.004 (1.001, 1.007)	0.999 (0.993, 1.005)	1.006 (0.995, 1.017)	1.002 (0.991, 1.013)	1.008 (0.998, 1.019)

^a ^Associations are expressed per IQR increase (listed in brackets in the first column). We used models with age as underlying time scale, stratified by sex and adjusted for marital status, region of origin, standardized household income, PC4 composite SES, mean income neighborhood, unemployment neighborhood, percentage of immigrants neighborhood, mean income region, unemployment region and percentage of immigrants region

^b ^Estimated with the average of the national LUR, Europe-wide hybrid and national dispersion models

^c ^Estimated with a national LUR model

Surrounding green (negatively) and air pollution (positively) were associated with the secondary outcomes cerebrovascular disease, COPD and dementia mortality (Table S3, Additional file 1). Of all air pollutants, only OP^DTT^ was associated with ischemic heart disease. Road-traffic noise was not associated with ischemic heart disease, cerebrovascular disease and COPD mortality and negatively associated with dementia mortality. Rail-traffic noise, on the other hand, was associated with dementia mortality. Spline analyses showed a threshold-shaped curve of rail-traffic noise with dementia mortality (Figure S1i, Additional file 1).

**Fig. 1:**
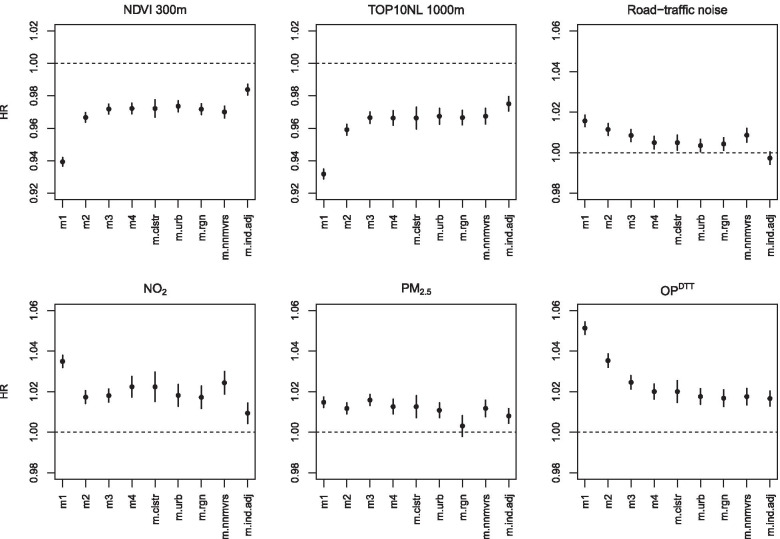
Associations of exposures with non-accidental mortality in a *priori* specified models ^a, b^. ^a^ Associations are expressed per IQR increase. Model 1 (m1) included the baseline hazard, a strata term for sex. Model 2 (m2) is additionally adjusted for standardized household income, region of origin and marital status. Model 3 (m3) is additionally adjusted for socio-economic composite score (based on the educational, occupational and economical status) at a four digit postal code level. Model 4 (m4, main model) is additionally adjusted for mean income per income recipient of the region, unemployment rate of the region, percentage non-western immigrants of the region, mean income per income recipient of the neighborhood, unemployment rate of the neighborhood and percentage non-western immigrants of the neighborhood. Sensitivity analysis: m.clstr (main model additionally included a cluster term for neighborhood code), m.urban: (main model additionally adjusted for degree of urbanization), m.region (main model additionally adjusted for region of the Netherlands), m.nnmvrs (main model for all subjects that did not move 5 years before the start of the follow-up period), m.ind.adj (main model indirectly adjusted for smoking status and BMI). ^b^ NO_2_ and PM_2.5_ were estimated with the average of the national LUR, Europe-wide hybrid and national dispersion models. OP^DTT^ was estimated with a national LUR model

Overall, associations of surrounding green, NO_2_, OP^DTT^ and road-traffic noise attenuated in models with increasing degree of adjustment for potential confounders (Fig. 1 for non-accidental mortality, Figure S3a-h for other mortality outcomes, Additional file 1). Associations with PM_2.5_ were less affected by adjustments for potential confounders. For neurodegenerative disease mortality, there was no clear pattern with increasing degree of adjustment for potential confounders. Sensitivity analysis showed that most associations were robust to the inclusion of a cluster for neighborhood and additional adjustment for degree of urbanization (Fig. 1 for non-accidental mortality, Figure S3a-h for other mortality outcomes, Additional file 1). Additional adjustment for large regions of the Netherlands generally attenuated associations with air pollution (especially PM_2.5_), but not with surrounding green and traffic noise. Associations from analyses restricted to all subjects that did not move in the 5 years prior to the follow-up period were similar to associations in the full cohort.

In our survey sample with lifestyle data, NDVI 300 m was weakly lower (3.8% of IQR) for current smokers compared to never smokers and for obese people (3.2% of IQR) compared to normal weight people (Table S4, Additional file 1). PM_2.5_ and OP^DTT^ concentrations were weakly higher for obese people compared to normal weight people, while concentrations were relatively similar for current, ex- and never-smokers. NDVI and air pollution exposure for the more numerous overweight adults were not different from normal weight subjects. For NO_2_, concentrations were higher (2.2% of IQR) for current smokers compared to ex- and never-smokers. Road-traffic noise levels were higher (3.5% of IQR) for current smokers compared to never-smokers in our population with lifestyle data. As a result of the relations between the exposures and BMI and smoking status, HRs of the exposures with non-accidental mortality attenuated after indirect adjustment for smoking status and BMI (Fig. 1). The degree of attenuation was least for PM_2.5_ and OP^DTT^, exposures not associated with smoking. Associations of surrounding green and air pollution with non-accidental mortality attenuated but remained significant, the already weak associations of road-traffic noise with non-accidental mortality disappeared.

**Fig. 2:**
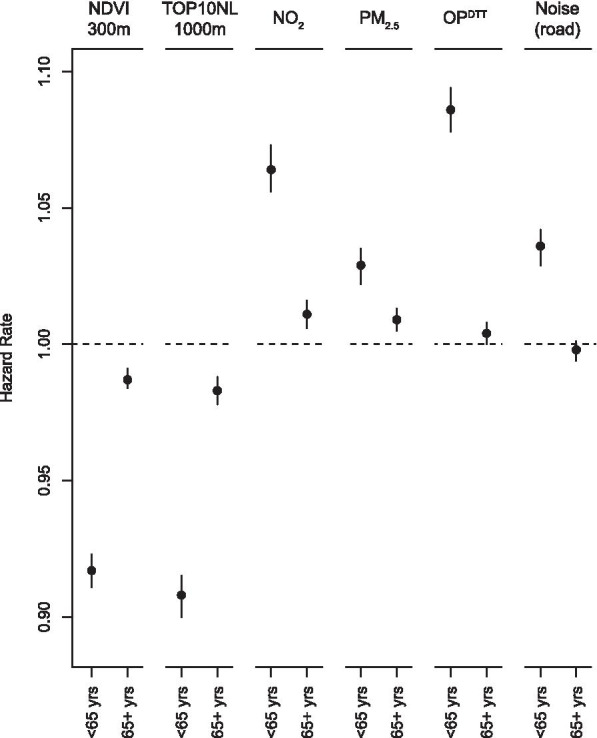
Associations of exposures with non-accidental mortality modified by age ^a, b^. ^a^ Associations are expressed per IQR increase. We used models with age as underlying time scale, stratified by sex and adjusted for marital status, region of origin, standardized household income, PC4 composite SES, mean income neighborhood, unemployment neighborhood, percentage of immigrants neighborhood, mean income region, unemployment region and percentage of immigrants region. ^b^ NO_2_ and PM_2.5_ were estimated with the average of the national LUR, Europe-wide hybrid and national dispersion models. OP^DTT^ was estimated with a national LUR model

In general, associations with surrounding green and air pollution were substantially stronger for the non-elderly than for the elderly (Fig. 2 for non-accidental mortality, Figure S4 for cause-specific mortality, Additional file 1). For the non-elderly, associations with surrounding green and air pollution were significant in the expected direction for non-accidental, circulatory disease, respiratory disease and lung cancer mortality. For the elderly, we found associations with respiratory disease and lung cancer mortality, but weak or non-significant associations for non-accidental and circulatory disease mortality. In addition, road-traffic noise was associated with non-accidental, circulatory, respiratory disease and lung cancer mortality in the non-elderly. For neurodegenerative disease and dementia mortality, associations with surrounding green were stronger for the non-elderly than for the elderly. For air pollution, associations were generally non-significant (neurodegenerative disease mortality) or stronger for the elderly (dementia mortality).

In general, associations of surrounding green with all mortality outcomes slightly attenuated after adjustment for air pollution (Table 3). For respiratory disease mortality, the HR of NDVI 300 m attenuated from 0.954 (95%CI: 0.943, 0.965) to 0.964 (95%CI: 0.952, 0.976) per IQR increase after adjustment for NO_2_. Associations of air pollutants with all mortality outcomes attenuated after adjustment for surrounding green, but generally remained statistically significant. The HR of NO_2_ attenuated from 1.022 (95% CI: 1.017, 1.028) to 1.008 (95% CI: 1.003, 1.014) for non-accidental mortality, after adjustment for NDVI 300 m. Associations of air pollution with respiratory disease and lung cancer mortality remained strongest from the evaluated causes of death. Road-traffic noise remained associated with lung cancer mortality after adjustment for surrounding green or air pollution. In a three-exposure model with NDVI 300 m, NO_2_ and road-traffic noise, associations of NDVI 300 m (HR = 0.934, 95% CI: 0.923, 0.947 per IQR increase), NO_2_ (HR = 1.026, 95% CI: 1.006, 1.047 per IQR increase) and road-traffic noise (HR = 1.013, 95% CI: 1.001, 1.025 per IQR increase) remained significant for lung cancer mortality. In a three-exposure model with NDVI 300 m, PM_2.5_ and road-traffic noise, associations of NDVI 300 m (HR = 0.933, 95%CI: 0.922, 0.945 per IQR increase), PM_2.5_ (HR = 1.026, 95% CI: 1.013, 1.040 per IQR increase) and road-traffic noise (HR = 1.014, 95% CI: 1.003, 1.026 per IQR increase) also remained significant.

**Table 3:** Associations of exposures with non-accidental and cause-specific mortality in two-exposure models ^a, b^

Model	Exposure (IQR)	Non-accidental mortality	Circulatory disease mortality	Respiratory disease mortality	Lung cancer mortality	Neurodegenerative disease mortality
		**HR (95% CI)**	**HR (95% CI)**	**HR (95% CI)**	**HR (95% CI)**	**HR (95% CI)**
NO2 + NDVI 300 m	NO2 (8.3)	1.008 (1.003, 1.014)	1.007 (0.997, 1.017)	1.040 (1.022, 1.059)	1.035 (1.017, 1.055)	0.977 (0.960, 0.994)
	NDVI 300 m (0.14)	0.974 (0.971, 0.978)	0.989 (0.982, 0.996)	0.964 (0.952, 0.976)	0.934 (0.922, 0.946)	0.972 (0.960, 0.983)
PM2.5 + NDVI 300 m	PM2.5 (1.4)	1.007 (1.003, 1.010)	1.014 (1.007, 1.021)	1.051 (1.038, 1.064)	1.030 (1.017, 1.043)	1.004 (0.992, 1.015)
	NDVI 300 m (0.14)	0.973 (0.970, 0.977)	0.990 (0.983, 0.997)	0.964 (0.952, 0.975)	0.931 (0.920, 0.943)	0.978 (0.967, 0.989)
OPDTT + NDVI 300 m	OPDTT (0.3)	1.009 (1.005, 1.013)	1.009 (1.001, 1.017)	1.048 (1.033, 1.062)	1.025 (1.011, 1.039)	1.010 (0.997, 1.023)
	NDVI 300 m (0.14)	0.976 (0.972, 0.979)	0.991 (0.983, 0.998)	0.972 (0.959, 0.984)	0.934 (0.922, 0.947)	0.982 (0.970, 0.994)
road-traffic noise + NDVI 300 m	road-traffic noise (7.5)	1.000 (0.997, 1.003)	1.003 (0.997, 1.009)	0.996 (0.985, 1.007)	1.019 (1.008, 1.031)	0.967 (0.957, 0.977)
	NDVI 300 m (0.14)	0.972 (0.969, 0.976)	0.988 (0.981, 0.994)	0.953 (0.942, 0.965)	0.929 (0.918, 0.941)	0.970 (0.959, 0.981)
NO2 + TOP10NL 1000 m	NO2 (8.3)	1.005 (1.000, 1.011)	1.004 (0.993, 1.015)	1.050 (1.030, 1.071)	1.053 (1.033, 1.074)	0.979 (0.961, 0.997)
	TOP10NL 1000 m (0.31)	0.969 (0.964, 0.974)	0.983 (0.973, 0.993)	0.981 (0.963, 0.999)	0.964 (0.947, 0.982)	0.974 (0.958, 0.991)
PM2.5 + TOP10NL 1000 m	PM2.5 (1.4)	1.007 (1.003, 1.011)	1.013 (1.006, 1.020)	1.055 (1.041, 1.068)	1.037 (1.023, 1.050)	1.006 (0.994, 1.018)
	TOP10NL 1000 m (0.31)	0.968 (0.964, 0.973)	0.985 (0.976, 0.994)	0.974 (0.959, 0.99)	0.952 (0.936, 0.968)	0.985 (0.971, 1.001)
OPDTT + TOP10NL 1000 m	OPDTT (0.3)	1.009 (1.005, 1.013)	1.008 (1.000, 1.016)	1.058 (1.043, 1.073)	1.041 (1.026, 1.056)	1.017 (1.003, 1.030)
	TOP10NL 1000 m (0.31)	0.972 (0.966, 0.977)	0.986 (0.976, 0.996)	0.992 (0.974, 1.010)	0.964 (0.947, 0.982)	0.994 (0.977, 1.011)
road-traffic noise + TOP10NL 1000 m	road-traffic noise (7.5)	1.001 (0.998, 1.004)	1.003 (0.997, 1.009)	0.999 (0.988, 1.010)	1.025 (1.014, 1.036)	0.969 (0.959, 0.979)
	TOP10NL 1000 m (0.31)	0.967 (0.962, 0.971)	0.982 (0.973, 0.991)	0.959 (0.943, 0.974)	0.948 (0.932, 0.963)	0.975 (0.960, 0.990)
NO2 + road-traffic noise	NO2 (8.3)	1.023 (1.018, 1.029)	1.012 (1.001, 1.023)	1.072 (1.053, 1.093)	1.063 (1.043, 1.083)	1.016 (0.998, 1.034)
	road-traffic noise (7.5)	0.999 (0.995, 1.002)	1.002 (0.995, 1.008)	0.985 (0.974, 0.997)	1.014 (1.002, 1.027)	0.968 (0.957, 0.979)
PM2.5 + road-traffic noise	PM2.5 (1.4)	1.012 (1.008, 1.016)	1.016 (1.008, 1.023)	1.062 (1.049, 1.076)	1.039 (1.026, 1.053)	1.018 (1.006, 1.031)
	road-traffic noise (7.5)	1.002 (0.999, 1.006)	1.002 (0.996, 1.008)	0.991 (0.980, 1.002)	1.023 (1.012, 1.035)	0.968 (0.958, 0.978)
OPDTT + road-traffic noise	OPDTT (0.3)	1.020 (1.016, 1.023)	1.012 (1.005, 1.020)	1.063 (1.050, 1.077)	1.050 (1.036, 1.064)	1.028 (1.015, 1.040)
	road-traffic noise (7.5)	1.002 (0.998, 1.005)	1.003 (0.997, 1.009)	0.994 (0.983, 1.004)	1.023 (1.012, 1.035)	0.967 (0.958, 0.977)

^a ^Associations are expressed per IQR increase. We used models with age as underlying time scale, stratified by sex and adjusted for marital status, region of origin, standardized household income, PC4 composite SES, mean income neighborhood, unemployment neighborhood, percentage of immigrants neighborhood, mean income region, unemployment region and percentage of immigrants region

^b ^NO_2_ and PM_2.5_ were with the average of the national LUR, Europe-wide hybrid and national dispersion models. OP^DTT^ was estimated with a national LUR model

Associations of surrounding green and air pollution with ischemic heart disease, cerebrovascular disease, COPD and dementia mortality also attenuated in two-exposure models (Table S5, Additional file 1). After adjustment for air pollution, TOP10NL 1000 m remained associated with cerebrovascular disease mortality, while NDVI 300 m was not associated anymore. NDVI 300 m remained associated with dementia mortality after adjustment for air pollution, while TOP10NL 1000 m was not associated anymore. Air pollution generally remained associated with cerebrovascular disease, COPD and dementia mortality after adjustment for surrounding green.

**Fig. 3:**
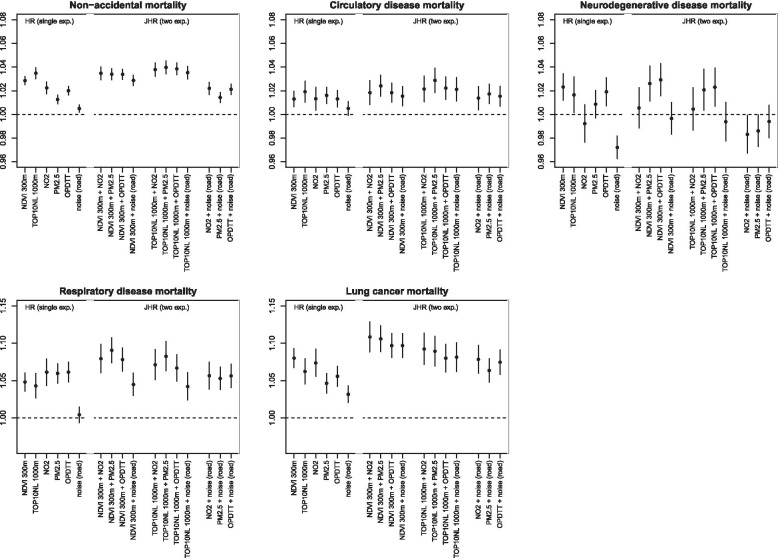
HRs and JHRs of associations of exposures with non-accidental and cause-specific mortality ^a, b, c^. ^a^ Associations are expressed per IQR increase. We used models with age as underlying time scale, stratified by sex and adjusted for marital status, region of origin, standardized household income, PC4 composite SES, mean income neighborhood, unemployment neighborhood, percentage of immigrants neighborhood, mean income region, unemployment region and percentage of immigrants region. ^b^ NO_2_ and PM_2.5_ were estimated with the average of the national LUR, Europe-wide hybrid and national dispersion models. OP^DTT^ was estimated with a national LUR model. ^c^ HRs and JHRs show associations to (a combination of) *decreased* surrounding green and *increased* air pollution and traffic noise

For non-accidental, circulatory disease, ischemic heart disease, cerebrovascular disease and neurodegenerative disease mortality, JHR of exposure to a combination of decreased surrounding green and increased air pollution concentrations were similar or only slightly higher than the HR from single-exposure models (Fig. 3 for main mortality outcomes, Figure S5 for secondary mortality outcomes, Additional file 1). For respiratory disease, COPD, lung cancer and dementia mortality, JHRs were larger than the HRs from single-exposure models. For respiratory disease mortality, the JHR for a combination of NDVI 300 m and PM_2.5_ was 1.091 (95% CI: 1.074, 1.107) and the HRs of NDVI 300 m and of PM_2.5_ from single-exposure models were 1.048 (95% CI: 1.036, 1.060) per IQR decrease and 1.060 (95% CI: 1.047, 1.073) per IQR increase, respectively.

## Discussion

Lower surrounding green and higher ambient air pollution were associated with an increased risk of death from several causes in a Dutch national cohort. In two-exposure models, associations of both exposures attenuated but remained significant for several mortality outcomes. Road-traffic noise was only associated with lung cancer mortality. In general, associations of all exposures with mortality were much stronger for the non-elderly than the elderly.

Surrounding green, air pollution and traffic noise have different underlying pathways to health effects. Surrounding green may promote physical activity and social interaction, decrease stress, and reduce exposure to air pollution and noise [44]. Exposure to air pollutants may lead to oxidative stress and systemic inflammation which in turn can result in cumulative or chronic effects, including mortality [3-5]. Exposure to traffic noise may induce stress and can result in reduced sleep quality, which in turn can adversely affect heart rate, blood pressure and changes in the metabolic system [45, 46].

Associations of surrounding green with non-accidental, circulatory disease, respiratory disease and lung cancer mortality reported in this study are in line with recent studies [14–16, 47]. Associations of NDVI with non-accidental, circulatory disease and respiratory disease mortality in our study were slightly weaker than HRs in other administrative cohorts [15, 16] and a meta-analyses of nine studies [48]. In the meta-analysis, the pooled HR for our IQR increment (0.14) was 0.94 (95% CI: 0.92, 0.96) for non-accidental mortality [48]. In single-exposure models, associations of surrounding green were somewhat stronger with respiratory disease and lung cancer mortality than with circulatory disease mortality. After adjustment for air pollution, associations of respiratory disease and lung cancer mortality with especially NDVI 300 m remained stronger. This is somewhat unexpected, as increased physical activity, increased social cohesion and reduced stress – potentially important pathways underlying effects of surrounding green—are likely more strongly related to circulatory diseases than to respiratory diseases and lung cancer mortality. Several other studies also reported stronger associations of surrounding green with respiratory disease mortality than with non-accidental or cardiovascular disease mortality [15, 16, 41, 49]. A possible explanation could be that surrounding green is a proxy for a person's exposure to natural airborne microbes. There is some evidence that exposure to certain levels of natural airborne microbes can cause health benefits by inhibiting the activities of interconnected cell signaling systems [50, 51]. However, our results should be carefully interpreted as we observed that current and ex-smokers had lower surrounding green levels compared to never-smokers in our population with lifestyle data and residual confounding by smoking status may have biased associations, especially with lung cancer and respiratory disease mortality.

Associations of air pollution with non-accidental and circulatory disease mortality were also smaller than associations with respiratory disease and lung cancer mortality. This is in line with other studies in Dutch populations [22, 24, 52], but not with recent meta-analyses of PM_2.5_ [3]. HRs in this study were expressed per IQR increase which, especially for PM_2.5_, were much smaller than the commonly used fixed increments in single-exposure studies. The HR for PM_2.5_ and non-accidental mortality expressed per 10 µg/m^3^ is 1.10 (single-exposure model), slightly higher than the combined HR of 1.08 in the most recent PM_2.5_ review [3]. Compared to HRs of recent meta-analyses for NO_2_ [4, 5], we found fairly similar associations of NO_2_ with non-accidental and circulatory disease mortality and stronger associations with respiratory disease and lung cancer mortality. Road-traffic noise was positively associated with lung cancer mortality, also after adjustment for air pollution and/or surrounding green. This could be due to stress related to noise exposure that may contribute to lung cancer incidence, but we also note that current smokers had higher road-traffic noise levels compared to never-smokers in our population with lifestyle data and thus residual confounding by smoking status may have biased associations.

Surrounding green, air pollution (only OP^DTT^) and rail-traffic noise were weakly associated with overall neurodegenerative disease mortality; road-traffic noise was not. Only a few studies evaluated associations of long-term exposure to surrounding green, air pollution or traffic noise with neurodegenerative disease or dementia mortality, and they generally found no associations [14, 18]. However, several studies reported associations of air pollution with dementia *incidence*, but not with road-traffic noise [6–8, 53–56]. Evidence for associations of environmental exposures with incidence of other neurodegenerative diseases, such as Parkinson’s disease and Alzheimer’s disease, is mixed [7, 9, 57–59]. Overall neurodegenerative mortality combined several diseases with very different etiology. Although approximately 63% of the neurodegenerative disease deaths were dementia deaths, we found that most air pollutants were associated with dementia mortality but not with overall neurodegenerative disease mortality. An important difference is that motor neuron disease, Parkinson’s disease, Alzheimer’s disease, Multiple sclerosis belong to the diseases of the nervous system (ICD-10 code: G), while dementia belongs to the mental and behavioral disorders (ICD-10 code: F). Dementia is not a specific disease but it is a group of symptoms caused by disorders that affect the brain, such as Alzheimer’s disease and stroke [60]. The difference in associations might indicate that exposure to air pollution may differently affect dementia and nervous system diseases, such as Parkinson’s and Alzheimer’s disease. Further, we acknowledge that mortality of neurodegenerative disease may be multiple years later than the diagnosis of the disease. While this is a limitation, the same applies to respiratory and circulatory disease mortality.

For almost all mortality outcomes, we found substantially stronger associations of surrounding green and air pollution in the non-elderly population than in the elderly population. Road-traffic noise was positively associated with non-accidental, circulatory and respiratory disease in the non-elderly population but not in the elderly population. Several other studies also reported stronger associations of surrounding green, air pollution or traffic noise with mortality in the younger age groups [12, 15, 22, 40, 41]. The difference is probably related to the many competing causes of death that could result in a higher baseline hazard in the elderly compared to the non-elderly population. This may translate into weaker HRs on the multiplicative scale for the elderly [61, 62]. Alternatively, the younger population might be more vulnerable for exposure to surrounding green, air pollution and traffic noise than the elderly.

HRs of associations of surrounding green and air pollution with non-accidental mortality attenuated but remained significant after indirect adjustment for smoking status and BMI, while the association with road-traffic noise was attenuated to null. The smallest attenuation was found for PM_2.5_ and OP^DTT^, consistent with PM_2.5_ and OP^DTT^ concentrations being similar for current, ex-, and never-smokers and higher only for obese (but not overweight) people compared to normal weight people. As the proportion of obese adults is relatively small compared to the proportion of overweight and smoking adults, the impact of adjustment in the full population may be small compared to exposures which are higher for current smokers or overweight adults.

We only performed formal indirect adjustment for non-accidental mortality. The impact of the indirect adjustment for lifestyle factors might differ between non-accidental and cause-specific mortality outcomes, as effects of smoking status and BMI likely differ between cause-specific mortality outcomes. Therefore the performed indirect adjustment based on associations between smoking status, BMI and non-accidental mortality may have under- or overestimated the potential bias for missing smoking and BMI for specific causes. Relations of BMI and smoking status with exposures and assumptions about effects of BMI and smoking provide some insight in the potential impact of the lack of adjustment for smoking and BMI. Effects of smoking are likely stronger for lung cancer and respiratory disease mortality compared to non-accidental mortality, while effects of obesity/overweight are likely stronger for circulatory disease mortality compared to non-accidental mortality and weaker for respiratory disease and lung cancer mortality. Hence, the indirect adjustment based on associations between smoking status, BMI and non-accidental mortality may not have fully accounted for residual confounding in the associations of PM_2.5_ and OP^DTT^ with circulatory disease mortality and probably overestimated residual confounding for respiratory disease and lung cancer mortality, as PM_2.5_ and OP^DTT^ concentrations were similar for current, ex-, and never-smokers, but higher for obese people compared to normal weight people. For these exposures, the pattern of higher HRs for respiratory and lung cancer mortality is thus unlikely due to unaccounted residual confounding. Road-traffic noise was higher for current smokers compared to never smokers, therefore the lack of adjustment for smoking status may have led to an overestimation of the association for road-traffic noise with lung cancer mortality, insufficiently characterized by the indirect adjustment for BMI and smoking status based on non-accidental mortality. As current smokers and obese people had lower NDVI 300 m and higher NO_2_ concentrations compared to never smokers and normal weight people, the lack of adjustment for lifestyle factors may have impacted associations of both exposures with circulatory disease, respiratory disease and lung cancer mortality in our study. In short, the pattern of higher effect estimates for respiratory and lung cancer mortality for most exposures may be related to residual confounding for surrounding green, NO_2_ and road-traffic noise but not PM_2.5_ and OP^DTT^.

Associations of surrounding green and air pollution generally attenuated but remained significant in two-exposure models. This indicates that both exposures may have independent effects on mortality and information about the risk of air pollution is partly contained by surrounding green estimates and vice versa. The attenuation of the HRs of two-exposure models compared to the HRs of single-exposure models was dependent on the mutual correlation of the exposures and the strength of the associations with the outcome. In general, associations of air pollution attenuated more after adjustment for surrounding green than associations of surrounding green after adjustment for air pollution. Other studies also reported that associations of surrounding green with mortality were robust to adjustment for air pollution [15, 16, 41, 47]. However, only Nieuwenhuijsen et al. reported associations of both exposures [47]. They also found that associations of air pollution attenuated more than associations of surrounding green in two-exposure models. Our epidemiological approach does not allow a clear judgement on whether relations between surrounding green and air pollution are causal, partly causal or non-causal. Hence, we do not know whether positive effects of surrounding green are because surrounding green reduces exposure to air pollution. Lower air pollution concentrations in green areas could be due to the absence of air pollution sources in green areas, the limitation of transmission of emissions and removal of air pollutants from the air by trees and other vegetation. Most studies that focused on air pollution or traffic noise did not adjust for surrounding green. Therefore, little is known about the impact of adjustment for surrounding green on associations of air pollution and noise.

JHRs of exposure to a combination of decreased surrounding green and increased air pollution were larger than the HRs of single-exposure models, for respiratory disease, COPD, lung cancer and dementia mortality. This indicates that the information about the risk of one exposure is only partly contained by the other correlated exposure. Therefore, the total effect of combined exposure to air pollution and decreased surrounding green is underestimated if only one of these exposures is taken into account. However, the effect of that specific exposure is overestimated if one uses the HR of the single-exposure model. These findings are in line with our previous findings for cardio-metabolic morbidity, mental and general health [19–21].

Strengths of this study include the longitudinal design and the large population size (~ 10.5 million adults). Furthermore, we were able to study effects of surrounding green, air pollution and traffic noise on several mortality outcomes. We used multiple indicators of surrounding green and air pollution exposure and all indicators were calculated at the address level. Moreover, all environmental exposures were assessed between 2009 and 2011, i.e., 2–4 years prior to the start of the follow-up period. Several studies showed that the spatial variation of surrounding green, air pollution and traffic noise exposure levels remain stable over periods of about 10 years in western countries [15, 16, 63, 64].

As we had residential LUR, dispersion and hybrid modelled NO_2_, PM_2.5_ and BC concentrations, we used the average concentration of these exposures. A previous study showed that despite strong correlations between LUR, dispersion and hybrid modelled NO_2_, PM_2.5_ and BC, associations with mortality outcomes can differ [24]. Further, correlations between surrounding green, traffic noise and NO_2_, PM_2.5_ and BC slightly differed between the exposure assessment models. For example, the spearman correlation of NDVI 300 m with LUR modelled NO_2_ was -0.54 and with dispersion modelled NO_2_ was -0.41. The spearman correlation of road-traffic noise with hybrid modelled PM_2.5_ was 0.17 and with dispersion modelled PM_2.5_ was 0.29. Therefore, the use of different exposure assessment models to predict air pollution may affect the degree of confounding by other exposures.

A limitation of our study is that we did not have data about personal lifestyle factors, such as smoking status and BMI. We indirectly adjusted for both factors for non-accidental mortality. HRs of the exposures attenuated but remained significant. We used a randomly stratified sample of the Public Health Monitor 2012 that was similar to our study population across important characteristics, which is an important aspect of the indirect adjustment method [37, 38]. Since we had no information about the associations of smoking status and BMI for cause-specific mortality outcomes, we were not able to indirectly adjust for smoking status and BMI.

## Conclusion

In general, surrounding green was associated with a lower risk of non-accidental and cause-specific mortality, while air pollution was associated with a higher risk of non-accidental and cause-specific mortality. Road-traffic noise was only positively associated with lung cancer mortality. In two-exposure models, associations of surrounding green and air pollution attenuated but generally remained. Studies including only one of these correlated exposures may overestimate the associations with mortality outcomes attributed to the studied exposure. Associations of environmental exposures with most mortality outcomes were stronger for the non-elderly population than for the elderly population.

## Supplementary Information

**Additional file 1: Table S1.** Descriptive statistics of sample of the complete study population and the stratified random sample of the Public health monitor 2012 used in the indirect adjustment method ^a^. **Table S2.** Associations of NO2, PM2.5 and BC based on the LUR, hybrid and dispersion model with non-accidental, circulatory disease, respiratory disease, lung cancer and neurodegenerative disease mortality in single-exposure models ^a^. **Table S3.** Associations of exposures with secondary mortality outcomes in single-exposure models ^a^. **Table S4**. Associations of smoking status and BMI with exposures ^a, b, c^. **Table S5.** Associations of exposures with secondary mortality outcomes in multi-exposure models ^a, b^. **Figure S1.** a-i. Estimated exposure−response curves (M4, solid lines) and 95% CIs (dashed lines) for mortality (df=3, density bars are shown on x−axis) ^a^. **Figure S2.** Spearman rho correlations between surrounding green, air pollution and traffic noise ^a^. **Figure S3.** **a-h**. Associations of surrounding green, air pollution and traffic noise with non-accidental mortality in a *priori* specified models with increasing degree of covariate adjustment and in sensitivity models ^a^. **Figure S4.** **a-b**. Associations of surrounding green, air pollution and traffic noise with circulatory disease, respiratory disease, lung cancer, neurodegenerative disease, ischemic heart disease, cerebrovascular disease, COPD and dementia mortality modified by age ^a, b^. **Figure S5.** HRs and JHRs of associations of exposure to (a combination of) decreased surrounding green and increased air pollution and traffic noise with ischemic heart disease mortality, cerebrovascular disease mortality, COPD mortality and dementia mortality ^a^.

## Data Availability

The data that support the findings of this study are available from Statistics Netherlands (longitudinal mortality registry, individual covariate data), IRAS (air pollution) and RIVM (air pollution, traffic noise, surrounding green). Restrictions apply to the availability of the data from Statistics Netherlands, which were used under license for the current study, and so are not publicly available. All our analyses were performed within strict privacy rules; that is, only researchers who received a signed permit were allowed to do analyses within a secured environment.
