# Supplementary material for: Effects of exposure to surrounding green, air pollution and traffic noise with non-accidental and cause-specific mortality in the Dutch national cohort
Source: Environ Health. 2021 Jul 14;20:82. doi: 10.1186/s12940-021-00769-0 (PMC8281461; doi:10.1186/s12940-021-00769-0)

**Additional file 1 of**

**Effects of exposure** **to surrounding green, air pollution and traffic noise with non-accidental and cause-specific mortality in the Dutch national cohort**

**Authors and affiliations:**

Jochem O. Klompmaker (jklompmaker@hsph.harvard.edu)

National Institute for Public Health and the Environment (RIVM), Bilthoven, The Netherlands.

Institute for Risk Assessment Sciences (IRAS), Utrecht University, Utrecht, The Netherlands.

Nicole A.H. Janssen (Nicole.Janssen@rivm.nl)

National Institute for Public Health and the Environment (RIVM), Bilthoven, The Netherlands.

Lizan D. Bloemsma (LIZAN.BLOEMSMA@CUANSCHUTZ.EDU)

National Institute for Public Health and the Environment (RIVM), Bilthoven, The Netherlands.

Institute for Risk Assessment Sciences (IRAS), Utrecht University, Utrecht, The Netherlands.

Marten Marra (marten.marra@rivm.nl)

National Institute for Public Health and the Environment (RIVM), Bilthoven, The Netherlands.

Erik Lebret (elebret@xs4all.nl)

National Institute for Public Health and the Environment (RIVM), Bilthoven, The Netherlands.

Institute for Risk Assessment Sciences (IRAS), Utrecht University, Utrecht, The Netherlands.

Ulrike Gehring (U.Gehring@uu.nl)

Institute for Risk Assessment Sciences (IRAS), Utrecht University, Utrecht, The Netherlands.

Gerard Hoek (g.hoek@uu.nl)

Institute for Risk Assessment Sciences (IRAS), Utrecht University, Utrecht, The Netherlands.

**Funding:** This research was carried out in, and funded by, the framework of RIVM Strategic Program (SPR; S/121004 HERACLES), in which expertise and innovative projects prepare RIVM to respond to future issues in health and sustainability.

**Competing interest:** The authors declare that they have no competing interests.

**Supplemental Tables**

Table S1. Descriptive statistics of sample of the complete study population and the stratified random sample of the Public health monitor 2012 used in the indirect adjustment method ^a^.

| **Covariate** | **Category** | **Study population  (n = 10,481,566)** | **Sample of PHM 2012  (n = 48,970)** |
| --- | --- | --- | --- |
|  |  | **Percentage or median (IQR)** | **Percentage or median (IQR)** |
| **Individual covariates** |  |  |  |
| -Age |  | 53 (23) | 53 (24) |
| -Sex | male | 48.8 | 48.8 |
|  | female | 51.2 | 51.2 |
| -Marital status | married | 60.9 | 64.3 |
|  | widowed | 7.8 | 7.6 |
|  | divorced | 10.9 | 10.3 |
|  | single | 20.3 | 17.8 |
| -Region of origin | Morocco | 1.6 | 1.3 |
|  | Turkey | 1.9 | 1.7 |
|  | Suriname | 1.9 | 1.8 |
|  | Antilles Netherlands | 0.6 | 0.7 |
|  | non-western | 3.1 | 3.4 |
|  | western | 9.8 | 9.7 |
|  | Dutch | 81.1 | 81.4 |
| -Standardized household income | <1% | 0.5 | 0.5 |
|  | 1-5% | 1.3 | 1.1 |
|  | 5-10% | 3.3 | 2.9 |
|  | 10-25% | 12.5 | 13.1 |
|  | 25-50% | 24.9 | 24.9 |
|  | 50-75% | 27.4 | 27.4 |
|  | 75-90% | 17.7 | 17.7 |
|  | 90-95% | 6.2 | 6.3 |
|  | 95-99% | 5.0 | 5.0 |
|  | >99% | 1.3 | 1.1 |
| **Area-level covariates** |  |  |  |
| -Composite SES 4 digit postal code | Based on education, income and paid occupation (year = ?) | 0.31 (1.33) | 0.32 (1.28) |
| -Mean income NBH | Mean income per income recipient *€ 1000 (year = 2006) | 29.3 (5.4) | 29.4 (5.1) |
| -Unemployment rate NBH | Number of people with income support per 1000 inhabitants of 15-64 years (year = 2006) | 25.0 (10.0) | 24.0 (10.0) |
| -Percentage non-western immigrants NBH | Percentage non-western immigrants (year = 2006) | 7.0 (11.0) | 7.0 (11.0) |
| -Mean income region | Mean income per income recipient *€ 1000 (year = 2006) | 34.1 (2.6) | 34.4 (2.7) |
| -Unemployment rate region | Number of people with income support per 1000 inhabitants of 15-64 years (year = 2006) | 24.5 (7.3) | 24.5 (8.1) |
| -Percentage non-western immigrants region | Percentage non-western immigrants (year = 2006) | 8.4 (5.9) | 8.6 (5.4) |

^a^ Of the stratified random sample of the Public health monitor 2012, about 1% was underweight, 39% was overweight, 14% was obese, 38% was former smoker and 22% was current smoker.

Table S2. Associations of NO2, PM2.5 and BC based on the LUR, hybrid and dispersion model with non-accidental, circulatory disease, respiratory disease, lung cancer and neurodegenerative disease mortality in single-exposure models ^a^.

| **Exposure (IQR)** | **Model** | **Non-accidental mortality** | **Circulatory disease mortality** | **Respiratory disease mortality** | **Lung cancer mortality** | **Neurodegenerative disease mortality** |
| --- | --- | --- | --- | --- | --- | --- |
|  |  | **HR (95% CI)** | **HR (95% CI)** | **HR (95% CI)** | **HR (95% CI)** | **HR (95% CI)** |
| NO2 (8.3) | LUR | 1.015 (1.011, 1.020) | 1.005 (0.996, 1.013) | 1.036 (1.020, 1.052) | 1.056 (1.040, 1.073) | 0.989 (0.974, 1.004) |
|  | Hybrid | 1.023 (1.019, 1.027) | 1.016 (1.008, 1.024) | 1.048 (1.033, 1.062) | 1.059 (1.045, 1.075) | 0.992 (0.979, 1.005) |
|  | Dispersion | 1.010 (1.006, 1.014) | 1.007 (0.999, 1.015) | 1.047 (1.033, 1.061) | 1.044 (1.030, 1.059) | 1.001 (0.988, 1.014) |
| BC (0.3) | LUR | 1.008 (1.004, 1.012) | 1.006 (0.998, 1.013) | 1.026 (1.012, 1.039) | 1.042 (1.028, 1.056) | 0.988 (0.976, 1.001) |
|  | Hybrid | 1.012 (1.009, 1.016) | 1.008 (1.001, 1.014) | 1.026 (1.015, 1.037) | 1.039 (1.027, 1.051) | 0.992 (0.982, 1.003) |
|  | Dispersion | 1.010 (1.007, 1.012) | 1.011 (1.005, 1.016) | 1.034 (1.024, 1.043) | 1.031 (1.021, 1.040) | 0.999 (0.990, 1.008) |
| PM2.5 (1.4) | LUR | 1.007 (1.001, 1.012) | 1.011 (1.002, 1.021) | 1.044 (1.026, 1.062) | 1.052 (1.033, 1.071) | 1.001 (0.985, 1.018) |
|  | Hybrid | 1.011 (1.008, 1.014) | 1.013 (1.007, 1.019) | 1.039 (1.029, 1.050) | 1.033 (1.022, 1.043) | 1.004 (0.995, 1.013) |
|  | Dispersion | 1.008 (1.005, 1.010) | 1.010 (1.005, 1.015) | 1.042 (1.034, 1.050) | 1.028 (1.019, 1.036) | 1.009 (1.001, 1.016) |

^a^ Associations are expressed per IQR increase of the average residential concentration of PM_2.5_, BC and NO_2_ of the three models. We used models with age as underlying time scale, stratified by sex and adjusted for marital status, region of origin, standardized household income, PC4 composite SES, mean income neighborhood, unemployment neighborhood, percentage of immigrants neighborhood, mean income region, unemployment region and percentage of immigrants region.

Table S3. Associations of exposures with secondary mortality outcomes in single-exposure models ^a^.

| **Exposure (IQR)** | **Ischemic heart disease mortality** | **Cerebrovascular disease mortality** | **COPD mortality** | **Dementia mortality** |
| --- | --- | --- | --- | --- |
|  | **HR (95% CI)** | **HR (95% CI)** | **HR (95% CI)** | **HR (95% CI)** |
| NDVI 300m (0.14) | 0.983 (0.970, 0.996) | 0.991 (0.978, 1.005) | 0.926 (0.911, 0.940) | 0.963 (0.950, 0.977) |
| TOP10NL 300m (0.23) | 0.979 (0.967, 0.991) | 0.980 (0.968, 0.993) | 0.933 (0.920, 0.947) | 0.987 (0.974, 1.000) |
| NDVI 1000m (0.14) | 0.997 (0.982, 1.012) | 0.988 (0.973, 1.004) | 0.962 (0.945, 0.979) | 0.969 (0.953, 0.985) |
| TOP10NL 1000m (0.31) | 0.981 (0.963, 0.998) | 0.964 (0.946, 0.982) | 0.940 (0.920, 0.961) | 0.980 (0.962, 0.999) |
| NO2 (8.3) ^b^ | 1.011 (0.992, 1.031) | 1.035 (1.015, 1.056) | 1.064 (1.040, 1.088) | 1.045 (1.024, 1.067) |
| PM2.5 (1.4) ^b^ | 1.009 (0.995, 1.023) | 1.026 (1.012, 1.041) | 1.059 (1.042, 1.077) | 1.066 (1.051, 1.082) |
| BC (0.3) ^b^ | 1.006 (0.991, 1.021) | 1.024 (1.009, 1.040) | 1.040 (1.022, 1.058) | 1.024 (1.008, 1.040) |
| PM10 (1.3) ^c^ | 0.995 (0.982, 1.008) | 1.005 (0.991, 1.018) | 1.011 (0.995, 1.026) | 0.981 (0.968, 0.995) |
| PMcoarse (0.8) ^c^ | 0.999 (0.986, 1.013) | 1.007 (0.994, 1.021) | 1.016 (1.000, 1.032) | 0.993 (0.979, 1.008) |
| OPDTT (0.3) ^c^ | 1.016 (1.002, 1.031) | 1.017 (1.002, 1.031) | 1.078 (1.060, 1.097) | 1.065 (1.050, 1.081) |
| OPESR (0.2) ^c^ | 1.008 (0.996, 1.021) | 1.015 (1.002, 1.028) | 1.024 (1.009, 1.039) | 1.012 (0.999, 1.026) |
| road-traffic noise (7.5) | 1.000 (0.988, 1.012) | 1.008 (0.996, 1.020) | 1.005 (0.991, 1.019) | 0.974 (0.962, 0.987) |
| rail-traffic noise (9.4) | 1.001 (0.988, 1.013) | 0.999 (0.986, 1.011) | 0.992 (0.978, 1.007) | 1.015 (1.002, 1.028) |

^a^ Associations are expressed per IQR increase. We used models with age as underlying time scale, stratified by sex and adjusted for marital status, region of origin, standardized household income, PC4 composite SES, mean income neighborhood, unemployment neighborhood, percentage of immigrants neighborhood, mean income region, unemployment region and percentage of immigrants region.

^b^ Estimated with the average of the national LUR, Europe-wide hybrid and national dispersion models

^c^ Estimated with a LUR model

Table S4. Associations of smoking status and BMI with exposures ^a, b, c^.

|  | **NDVI 300m** | | **TOP10NL 1000m** | | **NO_2_** | | **PM_2.5_** | | **OP^DTT^** | | **road-traffic noise** | |
| --- | --- | --- | --- | --- | --- | --- | --- | --- | --- | --- | --- | --- |
|  | **Beta (95% CI)** | **% of IQR** | **Beta (95% CI)** | **% of IQR** | **Beta (95% CI)** | **% of IQR** | **Beta (95% CI)** | **% of IQR** | **Beta (95% CI)** | **% of IQR** | **Beta (95% CI)** | **% of IQR** |
| **Ex-smoker** | -0.003 (-0.005, -0.001) | -2.1 | -0.006 (-0.009, -0.003) | -1.9 | 0.044 (-0.033, 0.121) | 0.5 | -0.009 (-0.026, 0.008) | -0.6 | 0.002 (-0.002, 0.005) | 0.6 | -0.010 (-0.126, 0.105) | -0.1 |
| **Current smoker** | -0.005 (-0.007, -0.003) | -3.8 | -0.007 (-0.010, -0.003) | -2.1 | 0.182 (0.092, 0.272) | 2.2 | 0.005 (-0.014, 0.024) | 0.4 | 0.000 (-0.004, 0.004) | 0.1 | 0.259 (0.125, 0.393) | 3.5 |
| **BMI <18.5 kg/m^2^** | 0.000 (-0.008, 0.008) | -0.1 | -0.014 (-0.028, 0.000) | -4.5 | 0.271 (-0.062, 0.605) | 3.3 | 0.062 (-0.01, 0.133) | 4.4 | 0.013 (-0.002, 0.028) | 4.8 | 0.516 (0.019, 1.013) | 6.9 |
| **BMI 25.0-29.9 kg/m^2^** | -0.001 (-0.003, 0.001) | -0.6 | 0.004 (0.001, 0.007) | 1.3 | -0.072 (-0.146, 0.001) | -0.9 | 0.002 (-0.014, 0.018) | 0.2 | 0.004 (0.000, 0.007) | 1,3 | -0.023 (-0.133, 0.087) | -0.3 |
| **BMI ≥30.0 kg/m^2^** | -0.004 (-0.007, -0.002) | -3.2 | 0.000 (-0.004, 0.005) | 0.1 | 0.029 (-0.073, 0.130) | 0.3 | 0.03 (0.008, 0.052) | 2.1 | 0.007 (0.003, 0.012) | 2.7 | 0.023 (-0.128, 0.174) | 0.3 |

^a^ Associations were estimated in a stratified random sample of the Public Health Monitor 2012 (n=48,970)

^b^ Reference levels were never smoker and BMI 18.5-24.9 kg/m^2^.

^c^ Models included smoking status and BMI simultaneously and were adjusted for sex, age, marital status, region of origin, standardized household income, PC4 composite SES, mean income neighborhood, unemployment neighborhood, percentage of immigrants neighborhood, mean income region, unemployment region and percentage of immigrants region.

Table S5. Associations of exposures with secondary mortality outcomes in multi-exposure models ^a, b^.

| **Model** | **Exposure (IQR)** | **Ischemic heart disease mortality** | **Cerebrovascular disease mortality** | **COPD mortality** | **Dementia mortality** |
| --- | --- | --- | --- | --- | --- |
|  |  | **HR (95% CI)** | **HR (95% CI)** | **HR (95% CI)** | **HR (95% CI)** |
| NO2 +  NDVI 300m | NO2 (8.3) | 1.002 (0.981, 1.023) | 1.035 (1.014, 1.057) | 1.025 (1.000, 1.050) | 1.028 (1.006, 1.051) |
|  | NDVI 300m (0.14) | 0.983 (0.969, 0.998) | 1.000 (0.986, 1.015) | 0.931 (0.916, 0.947) | 0.970 (0.955, 0.985) |
| PM2.5 +  NDVI 300m | PM2.5 (1.4) | 1.005 (0.991, 1.019) | 1.025 (1.010, 1.040) | 1.044 (1.026, 1.061) | 1.060 (1.044, 1.076) |
|  | NDVI 300m (0.14) | 0.984 (0.971, 0.997) | 0.997 (0.983, 1.010) | 0.933 (0.918, 0.949) | 0.974 (0.960, 0.989) |
| OPDTT +  NDVI 300m | OPDTT (0.3) | 1.010 (0.994, 1.026) | 1.016 (1.000, 1.032) | 1.050 (1.031, 1.070) | 1.058 (1.041, 1.076) |
|  | NDVI 300m (0.14) | 0.987 (0.972, 1.002) | 0.998 (0.983, 1.013) | 0.943 (0.927, 0.959) | 0.986 (0.971, 1.002) |
| road-traffic noise +  NDVI 300m | road-traffic noise (0.3) | 0.997 (0.985, 1.010) | 1.007 (0.994, 1.019) | 0.992 (0.977, 1.006) | 0.967 (0.954, 0.979) |
|  | NDVI 300m (0.14) | 0.982 (0.969, 0.996) | 0.993 (0.979, 1.007) | 0.924 (0.909, 0.939) | 0.956 (0.942, 0.970) |
| NO2 +  TOP10NL 1000m | NO2 (8.3) | 1.001 (0.979, 1.024) | 1.020 (0.997, 1.043) | 1.040 (1.014, 1.067) | 1.045 (1.021, 1.070) |
|  | TOP10NL 1000m (0.31) | 0.981 (0.961, 1.002) | 0.973 (0.952, 0.993) | 0.957 (0.934, 0.981) | 1.000 (0.978, 1.022) |
| PM2.5 +  TOP10NL 1000m | PM2.5 (1.4) | 1.005 (0.991, 1.020) | 1.020 (1.006, 1.035) | 1.050 (1.033, 1.068) | 1.066 (1.050, 1.082) |
|  | TOP10NL 1000m (0.31) | 0.982 (0.964, 1.000) | 0.970 (0.952, 0.988) | 0.954 (0.934, 0.976) | 0.999 (0.979, 1.018) |
| OPDTT +  TOP10NL 1000m | OPDTT (0.3) | 1.011 (0.995, 1.028) | 1.003 (0.987, 1.020) | 1.069 (1.049, 1.090) | 1.076 (1.057, 1.094) |
|  | TOP10NL 1000m (0.31) | 0.987 (0.967, 1.008) | 0.966 (0.946, 0.986) | 0.978 (0.955, 1.002) | 1.025 (1.003, 1.048) |
| road-traffic noise +  TOP10NL 1000m | road-traffic noise (0.3) | 0.998 (0.985, 1.010) | 1.003 (0.991, 1.016) | 0.997 (0.983, 1.011) | 0.971 (0.959, 0.983) |
|  | TOP10NL 1000m (0.31) | 0.980 (0.962, 0.998) | 0.965 (0.947, 0.983) | 0.94 (0.919, 0.96) | 0.972 (0.953, 0.991) |
| NO2 +  road-traffic noise | NO2 (8.3) | 1.014 (0.992, 1.036) | 1.036 (1.014, 1.059) | 1.075 (1.049, 1.103) | 1.082 (1.058, 1.107) |
|  | road-traffic noise (0.3) | 0.997 (0.983, 1.010) | 0.998 (0.985, 1.012) | 0.985 (0.969, 1.001) | 0.954 (0.941, 0.967) |
| PM2.5 +  road-traffic noise | PM2.5 (1.4) | 1.009 (0.995, 1.024) | 1.025 (1.010, 1.041) | 1.062 (1.044, 1.080) | 1.080 (1.064, 1.097) |
|  | road-traffic noise (0.3) | 0.998 (0.986, 1.011) | 1.002 (0.990, 1.015) | 0.992 (0.977, 1.006) | 0.958 (0.946, 0.971) |
| OPDTT +  road-traffic noise | OPDTT (0.3) | 1.017 (1.002, 1.032) | 1.015 (1.001, 1.030) | 1.080 (1.062, 1.099) | 1.076 (1.060, 1.093) |
|  | road-traffic noise (0.3) | 0.997 (0.985, 1.010) | 1.005 (0.993, 1.018) | 0.992 (0.977, 1.006) | 0.962 (0.949, 0.974) |

^a^ Associations are expressed per IQR increase. We used models with age as underlying time scale, stratified by sex and adjusted for marital status, region of origin, standardized household income, PC4 composite SES, mean income neighborhood, unemployment neighborhood, percentage of immigrants neighborhood, mean income region, unemployment region and percentage of immigrants region.

^b^ NO_2_ and PM_2.5_ were estimated with the average of the national LUR, Europe-wide hybrid and national dispersion models. OP^DTT^ was estimated with a LUR model.

**Supplemental Figure legends**

Figure S1a-i. Estimated exposure−response curves (M4, solid lines) and 95% CIs (dashed lines) for mortality (df=3, density bars are shown on x−axis) ^a^.

^a^ We used models with age as underlying time scale, stratified by sex and adjusted for marital status, region of origin, standardized household income, PC4 composite SES, mean income neighborhood, unemployment neighborhood, percentage of immigrants neighborhood, mean income region, unemployment region and percentage of immigrants region.

Figure S2. Spearman rho correlations between surrounding green, air pollution and traffic noise ^a^.

Figure S3a-h. Associations of surrounding green, air pollution and traffic noise with non-accidental mortality in a *priori* specified models with increasing degree of covariate adjustment and in sensitivity models ^a^.

^a^ Associations are expressed per IQR increase. Model 1 (m1) included the baseline hazard, a strata term for sex. Model 2 (m2) is additionally adjusted for standardized household income, region of origin and marital status. Model 3 (m3) is additionally adjusted for socio-economic composite score (based on the educational, occupational and economical status) at a four digit postal code level. Model 4 (m4, main model) is additionally adjusted for mean income per income recipient of the region, unemployment rate of the region, percentage non-western immigrants of the region, mean income per income recipient of the neighborhood, unemployment rate of the neighborhood and percentage non-western immigrants of the neighborhood. Sensitivity analysis (in grey): m.clstr (main model additionally included a cluster term for neighborhood code), m.urban: (main model additionally adjusted for degree of urbanization), m.region (main model additionally adjusted for region of the Netherlands), m.nnmvrs (main model for all subjects that did not move 5 years before the start of the follow-up period.

Figure S4a-b. Associations of surrounding green, air pollution and traffic noise with circulatory disease, respiratory disease, lung cancer, neurodegenerative disease, ischemic heart disease, cerebrovascular disease, COPD and dementia mortality modified by age ^a, b^.

^a^ Associations are expressed per IQR increase. We used models with age as underlying time scale, stratified by sex and adjusted for marital status, region of origin, standardized household income, PC4 composite SES, mean income neighborhood, unemployment neighborhood, percentage of immigrants neighborhood, mean income region, unemployment region and percentage of immigrants region.

^b^ NO_2_ and PM_2.5_ were estimated with the average of the national LUR, Europe-wide hybrid and national dispersion models. OP^DTT^ was estimated with a LUR model.

Figure S5. HRs and JHRs of associations of exposure to (a combination of) decreased surrounding green and increased air pollution and traffic noise with ischemic heart disease mortality, cerebrovascular disease mortality, COPD mortality and dementia mortality ^a^.

^a^ We used models with age as underlying time scale, stratified by sex and adjusted for marital status, region of origin, standardized household income, PC4 composite SES, mean income neighborhood, unemployment neighborhood, percentage of immigrants neighborhood, mean income region, unemployment region and percentage of immigrants region.


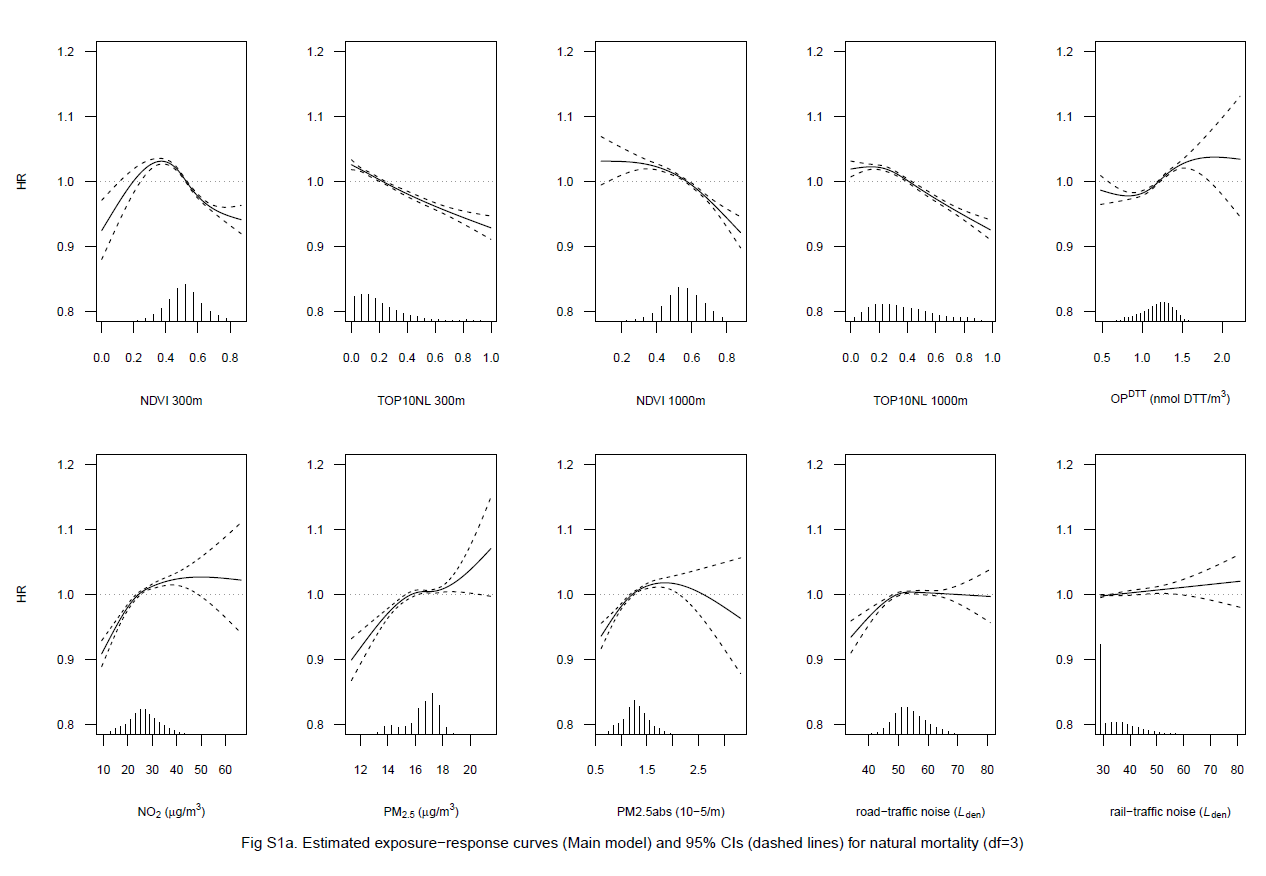


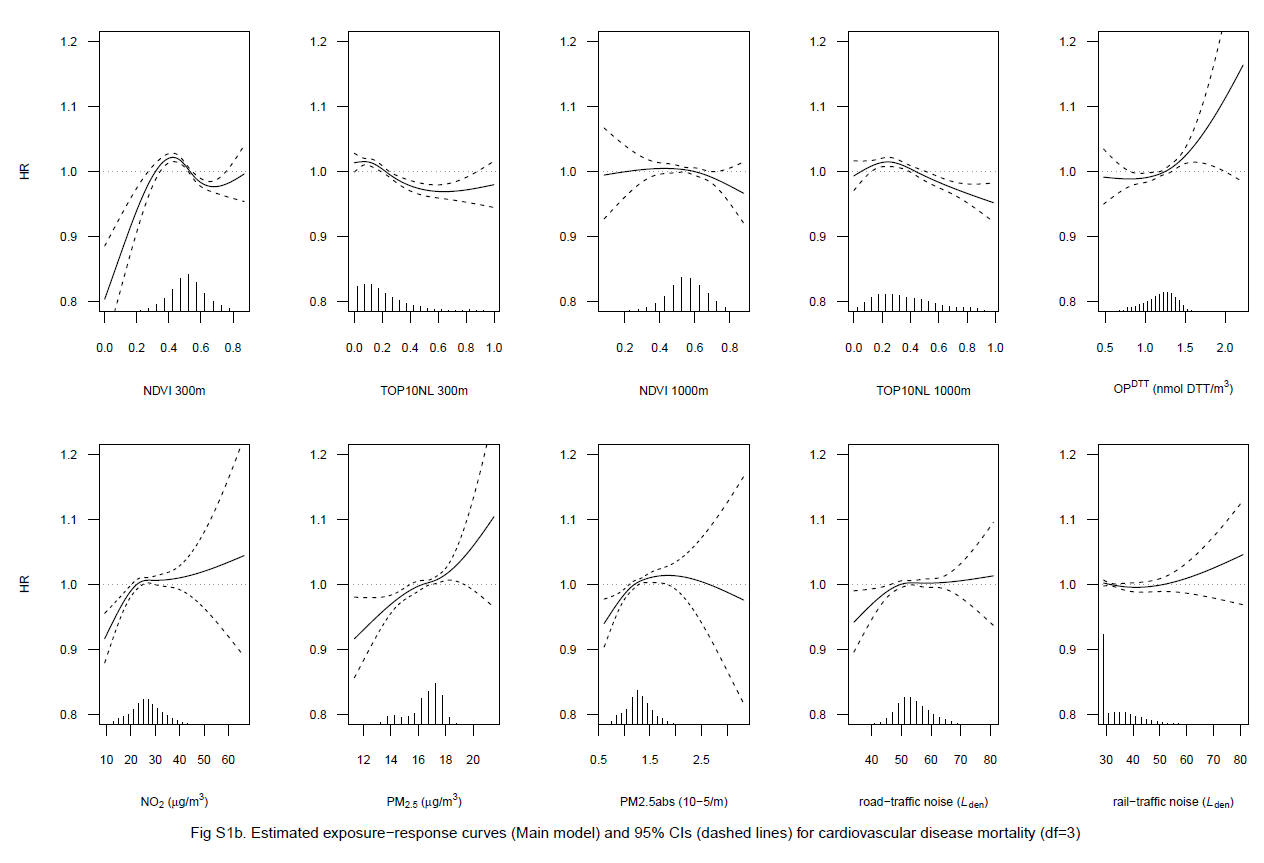


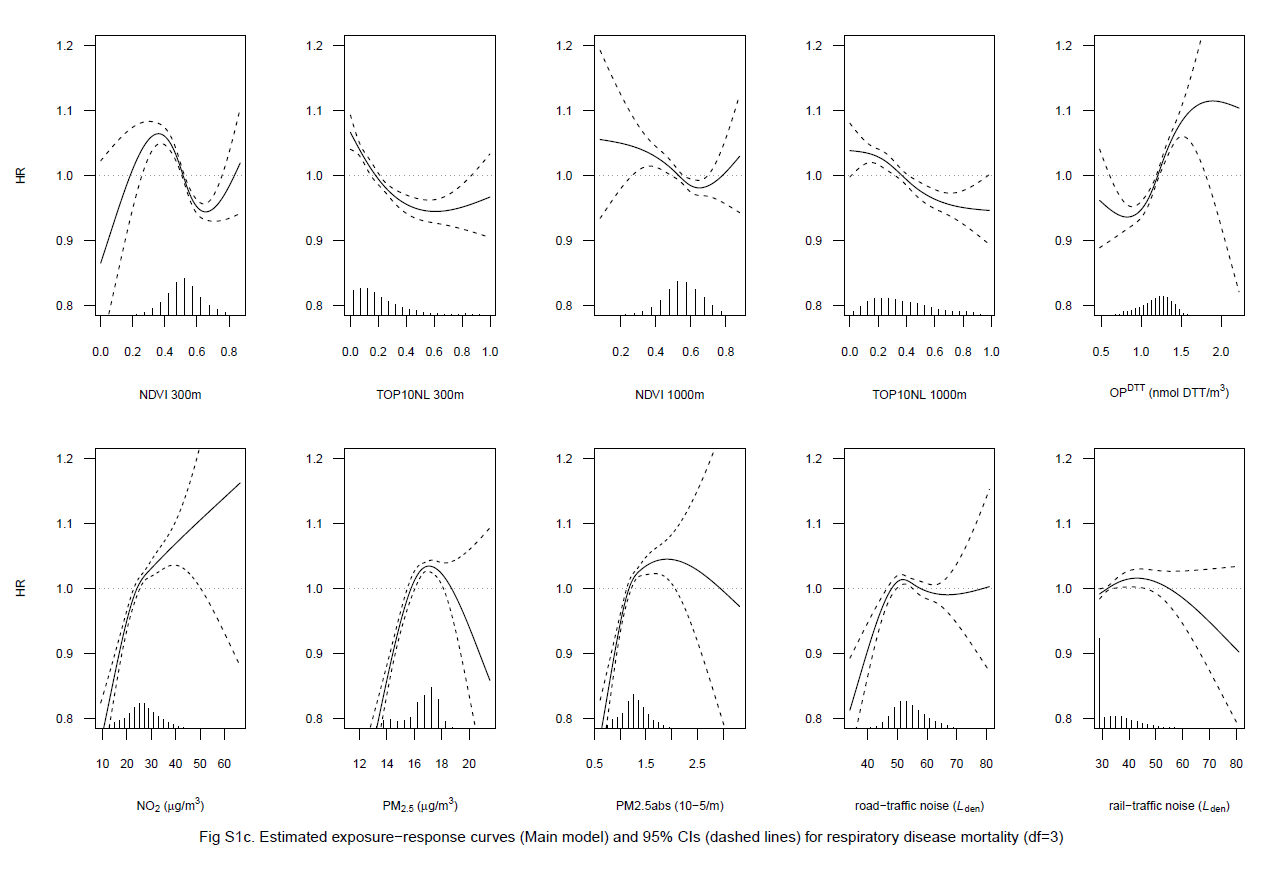


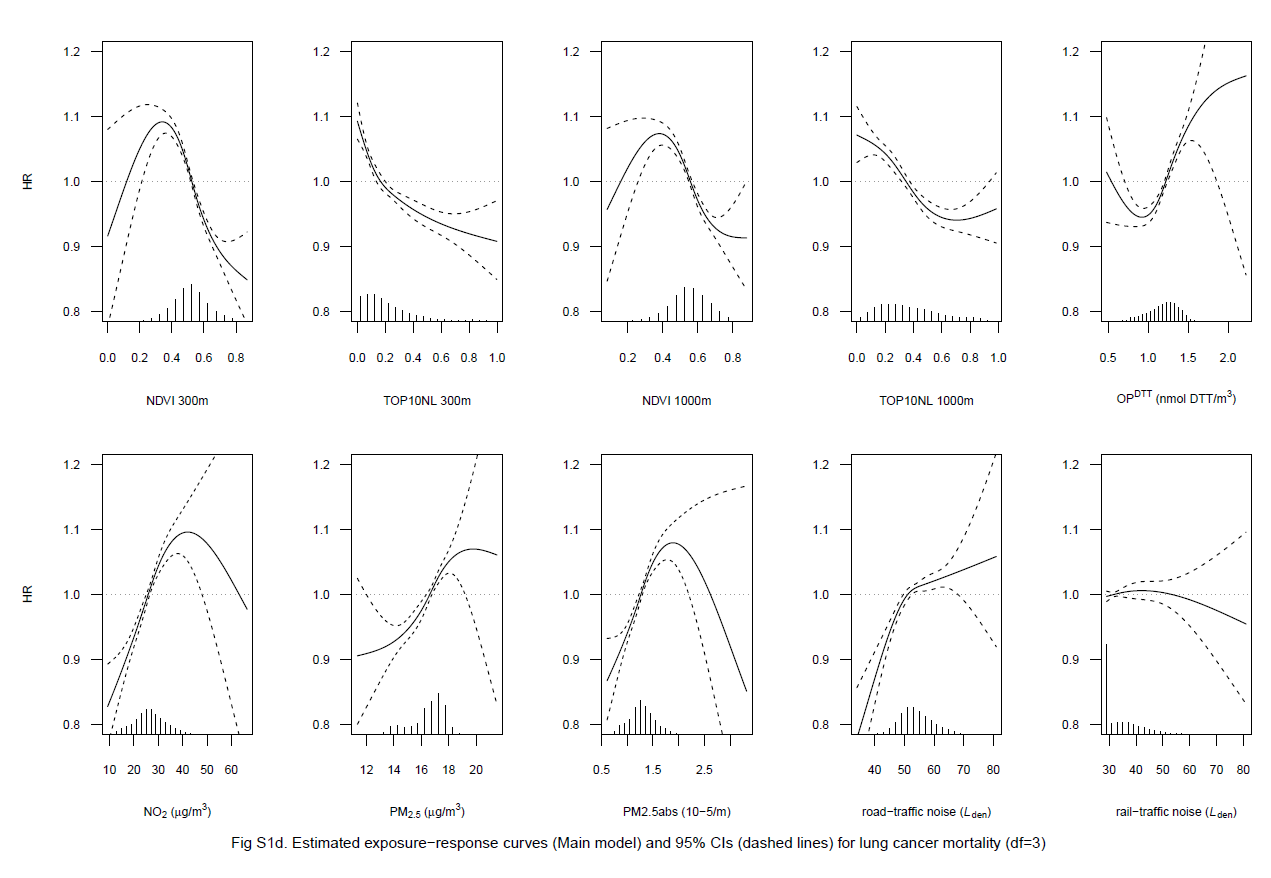


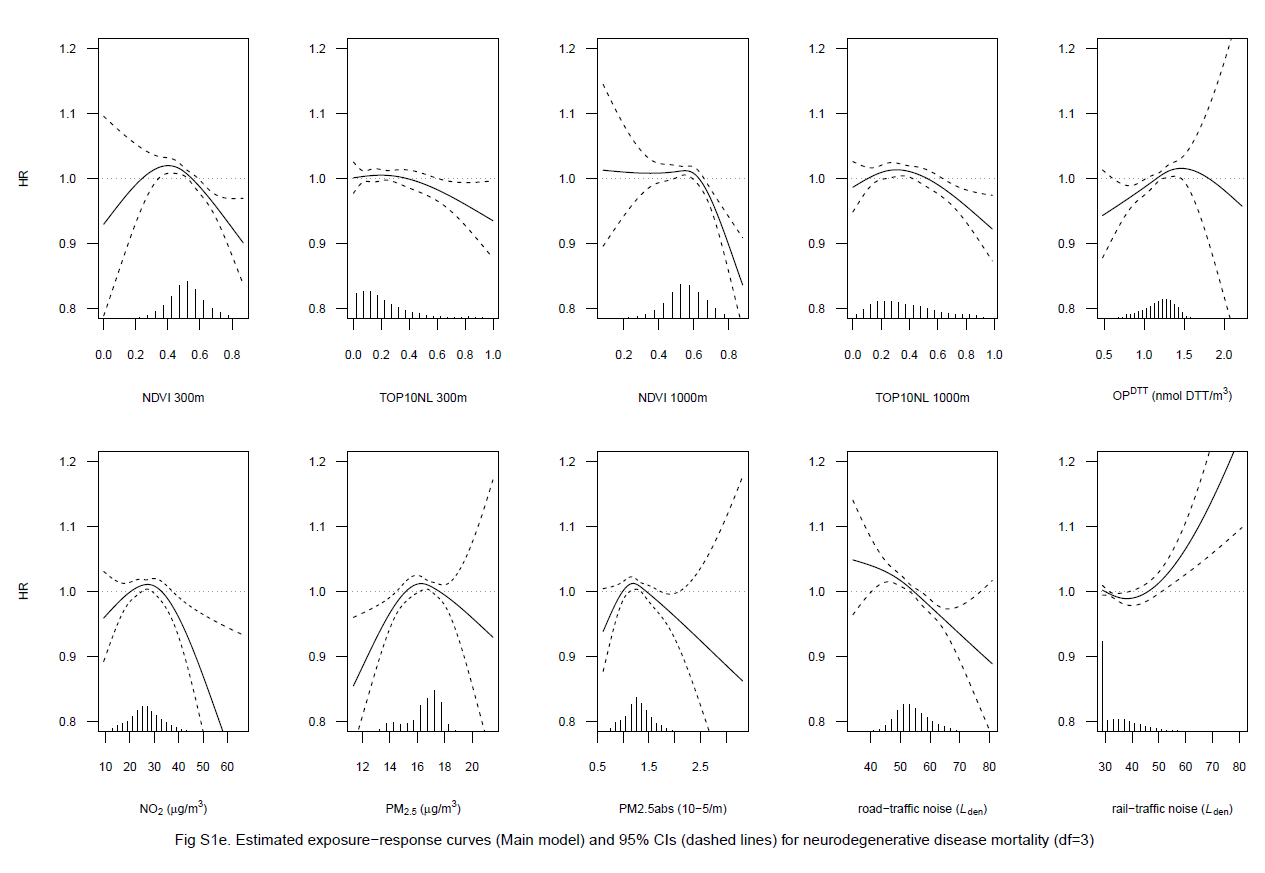


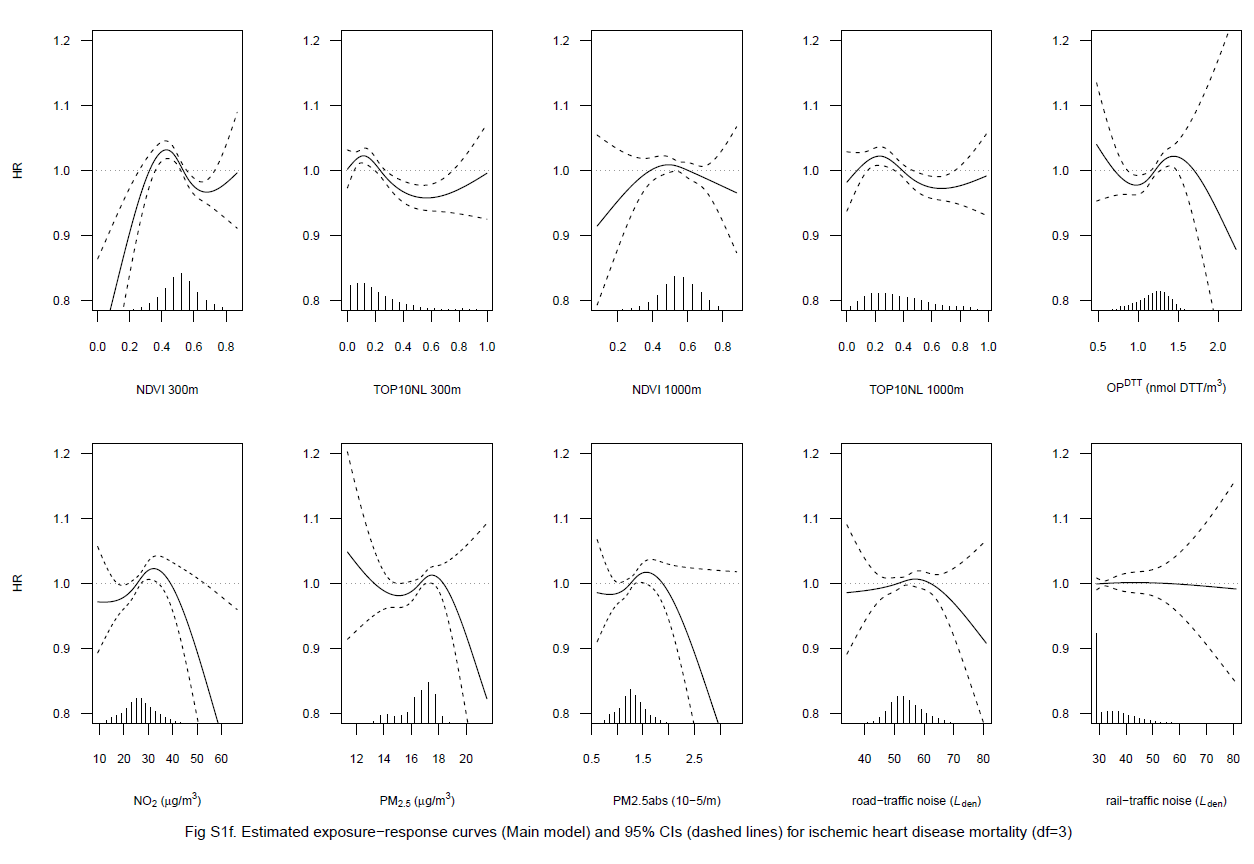


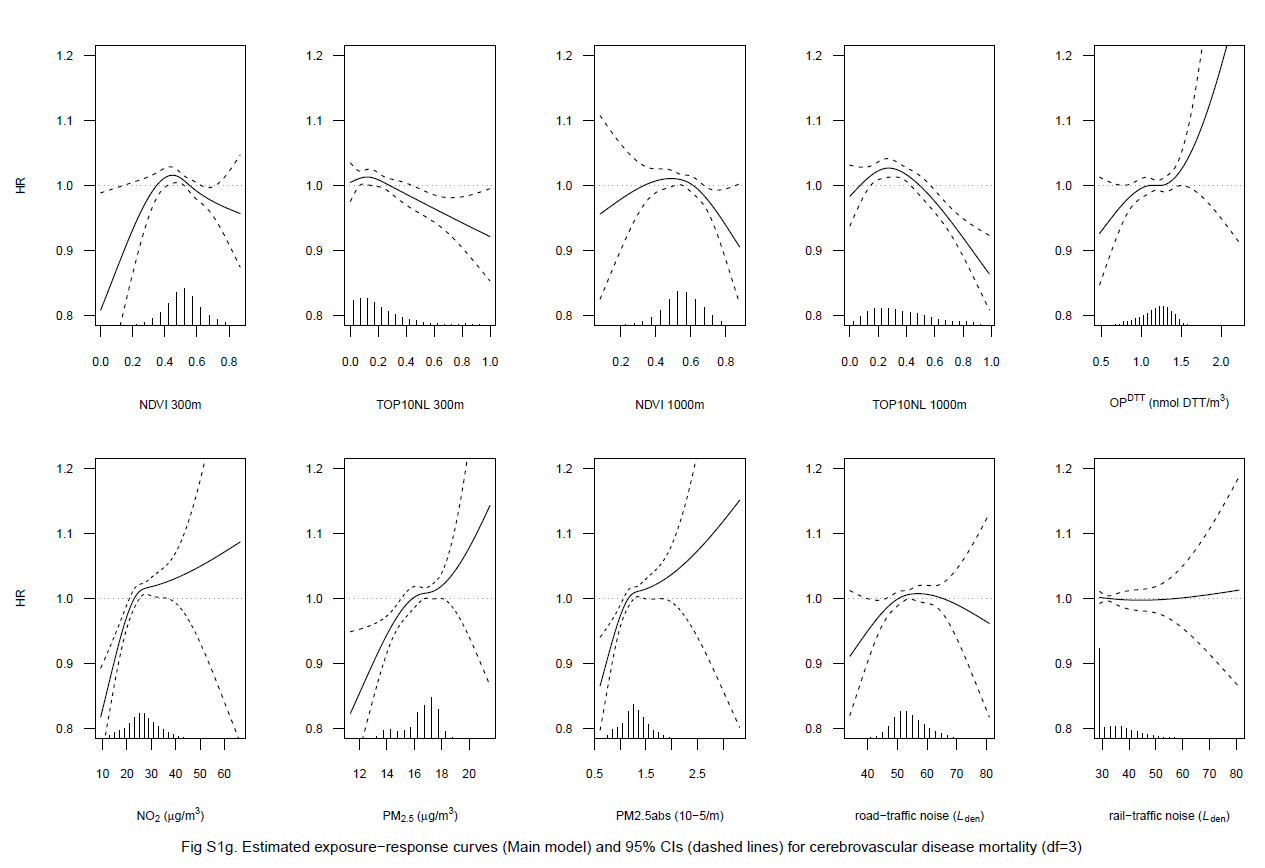


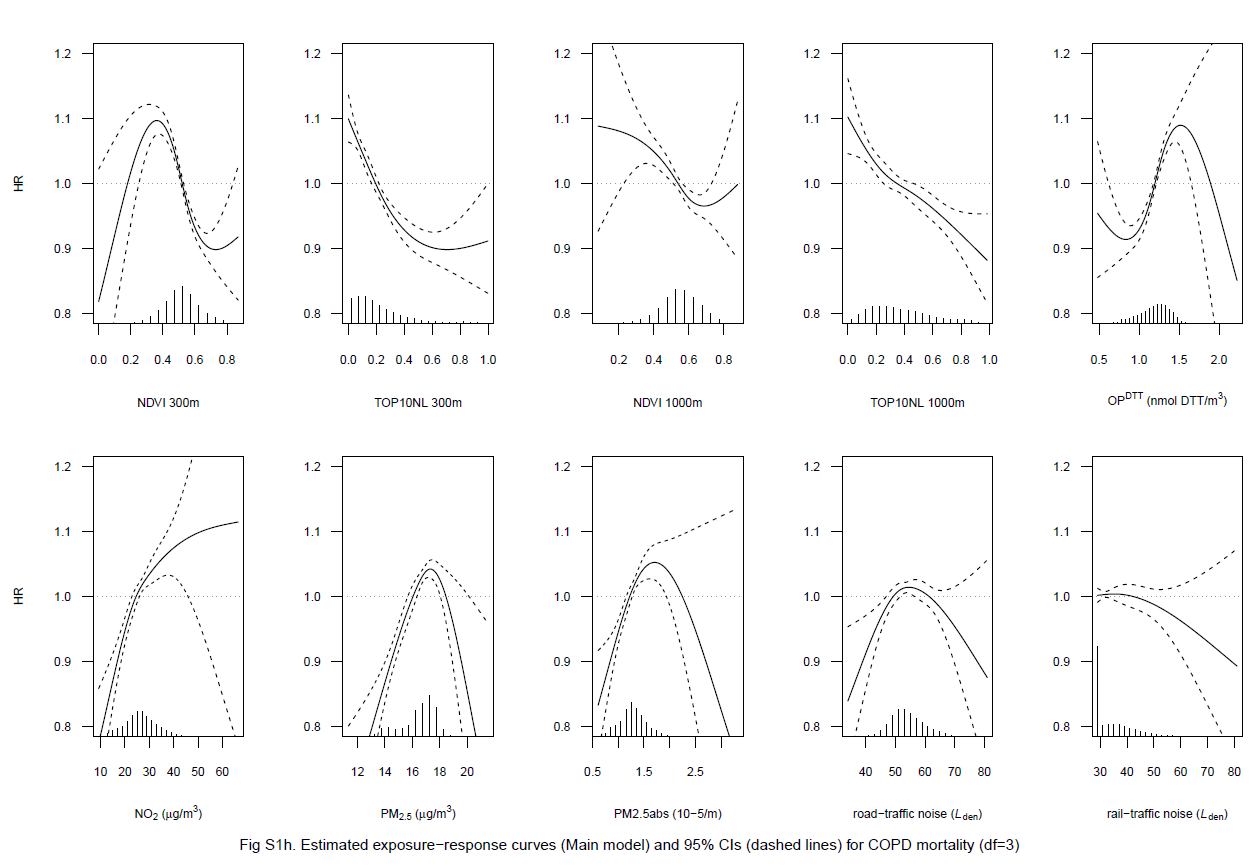


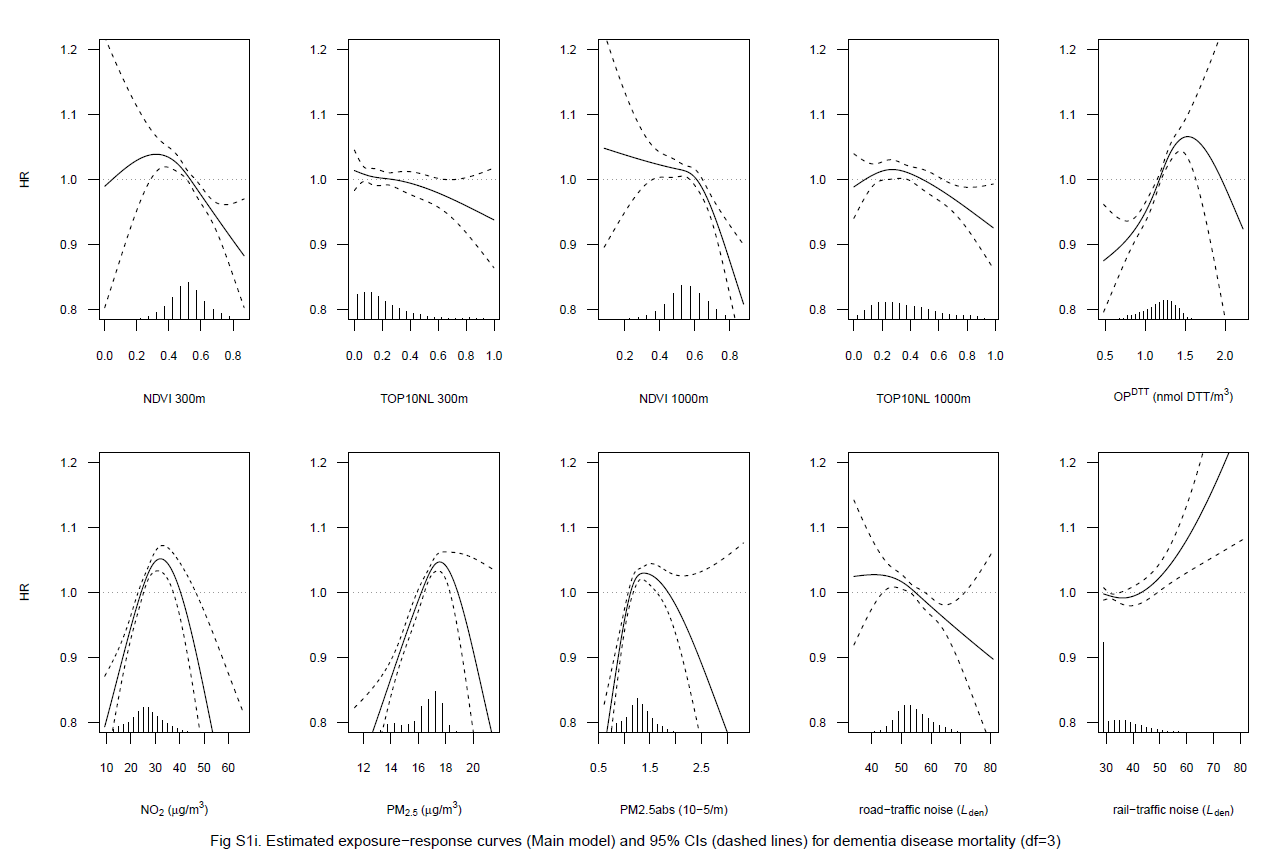


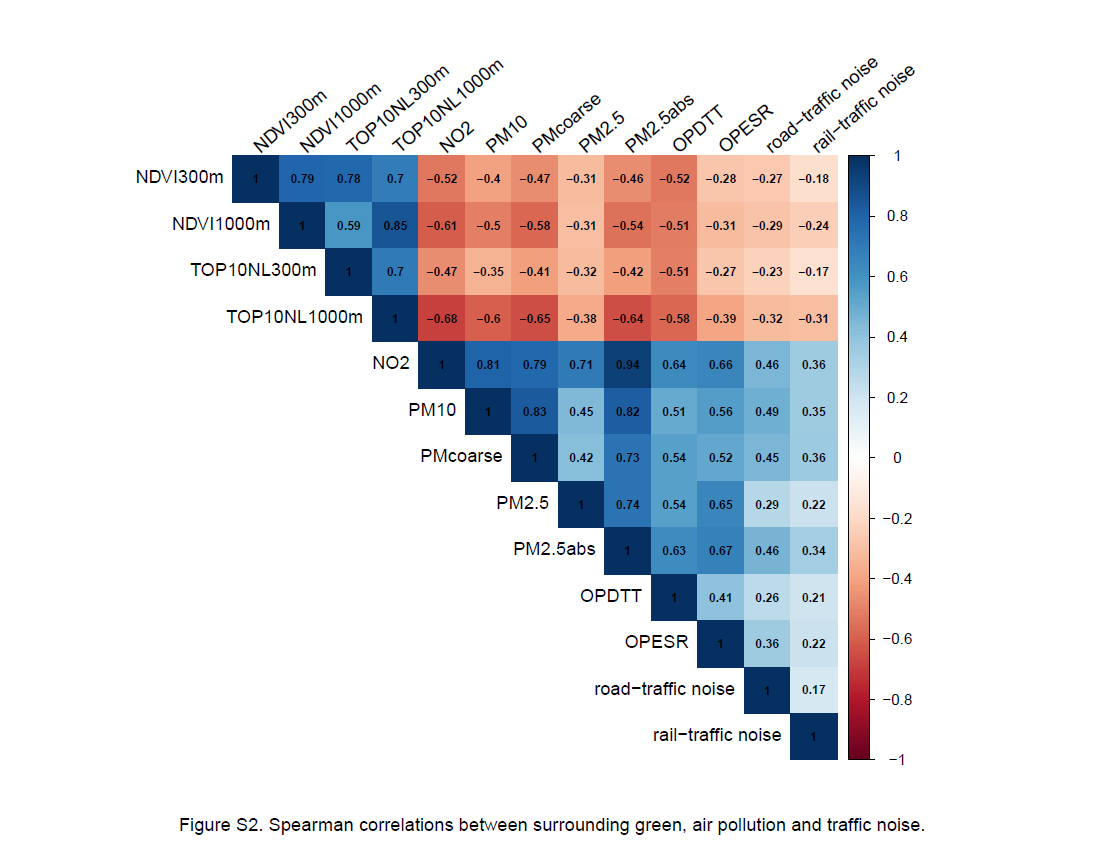


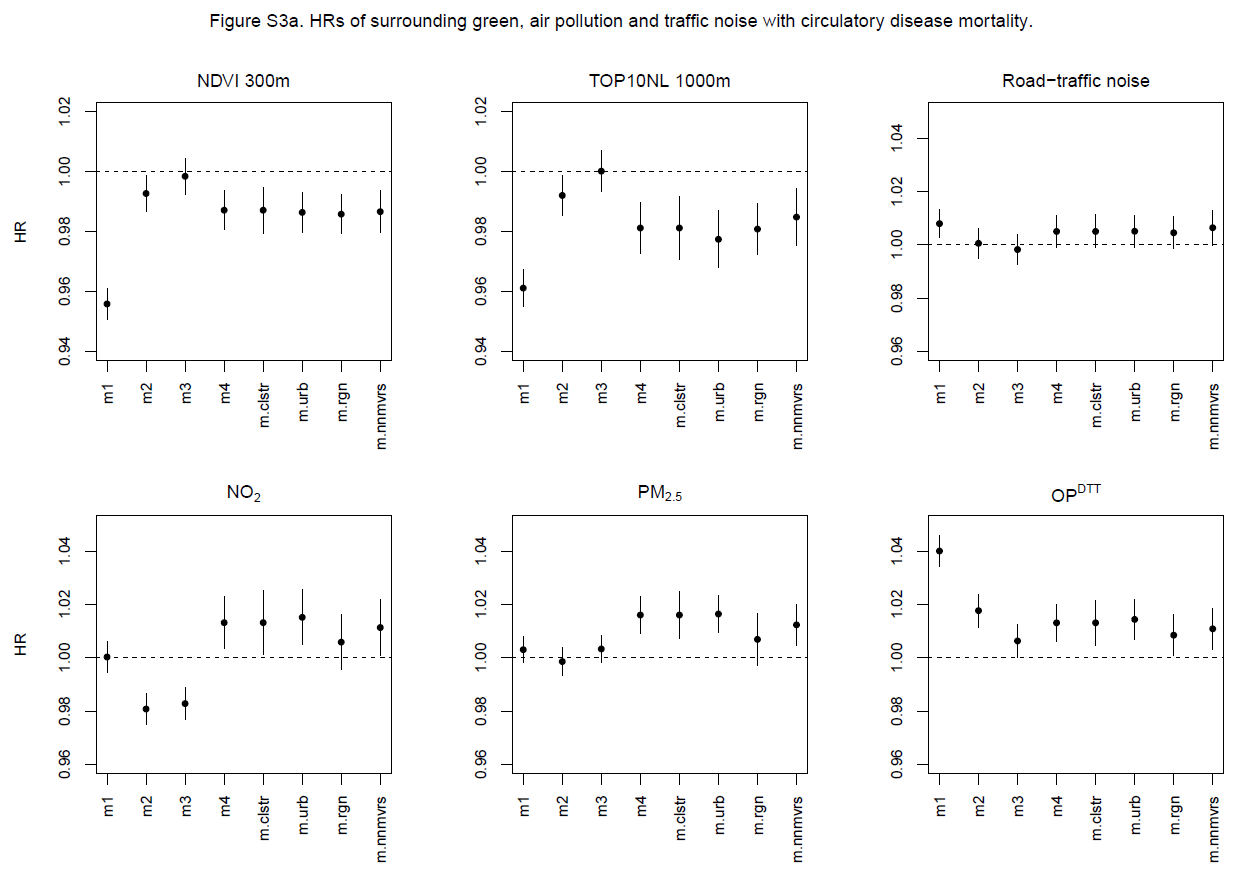


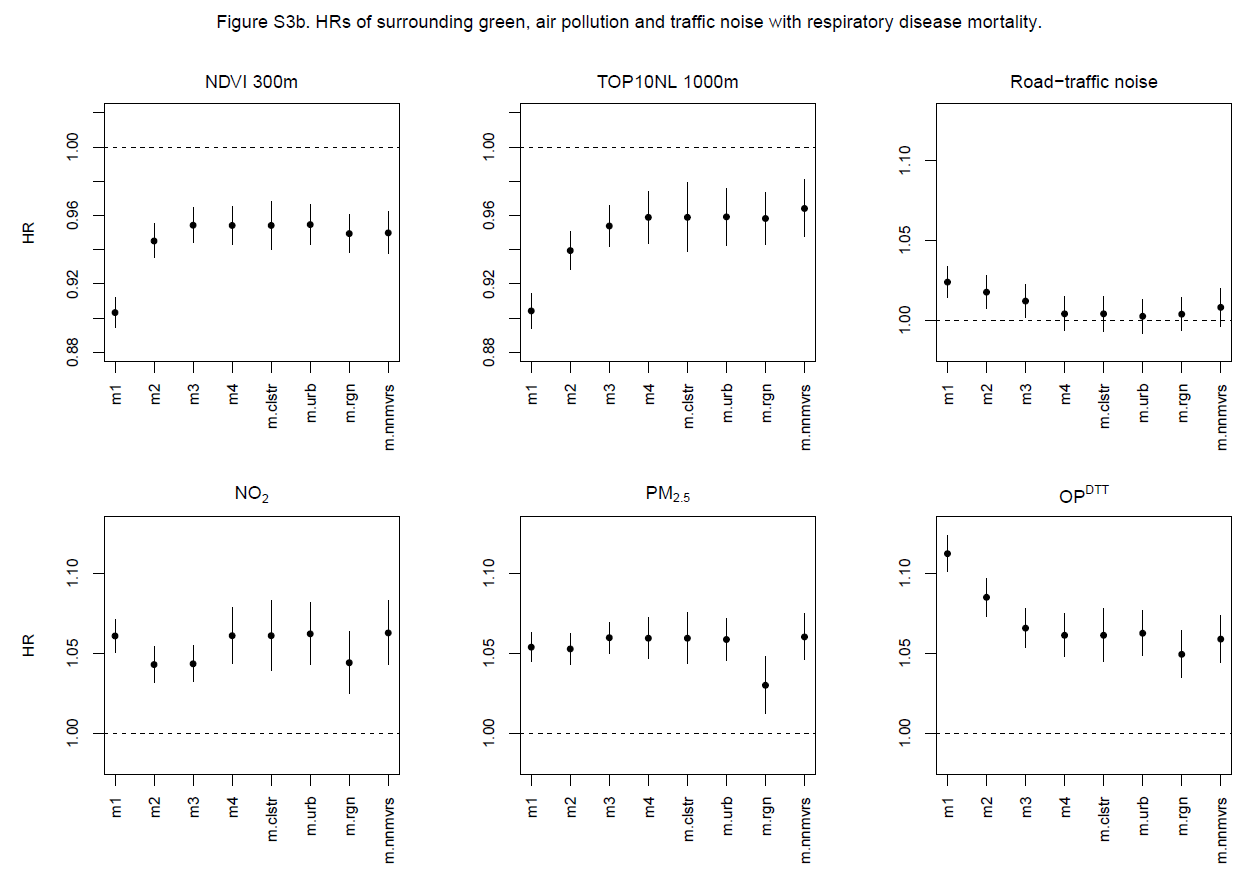


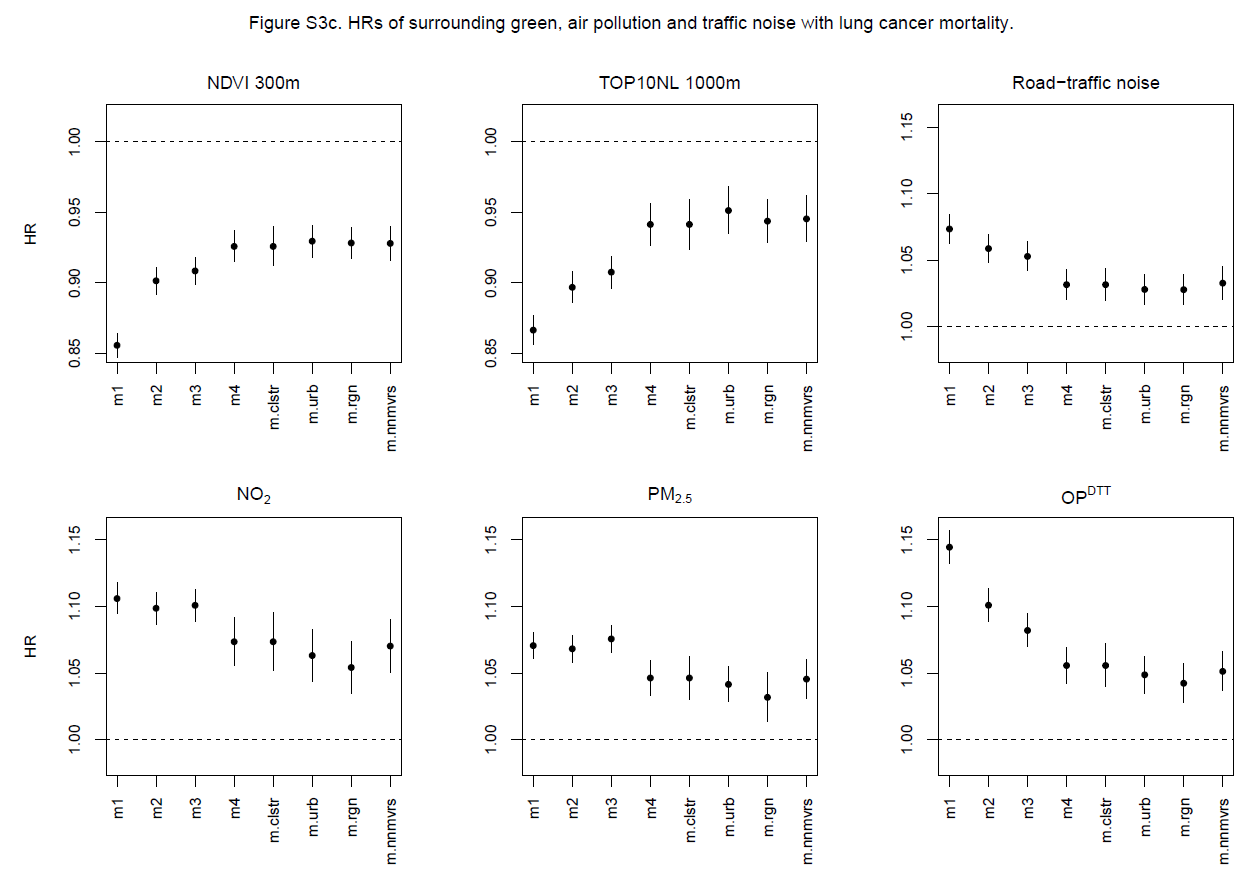


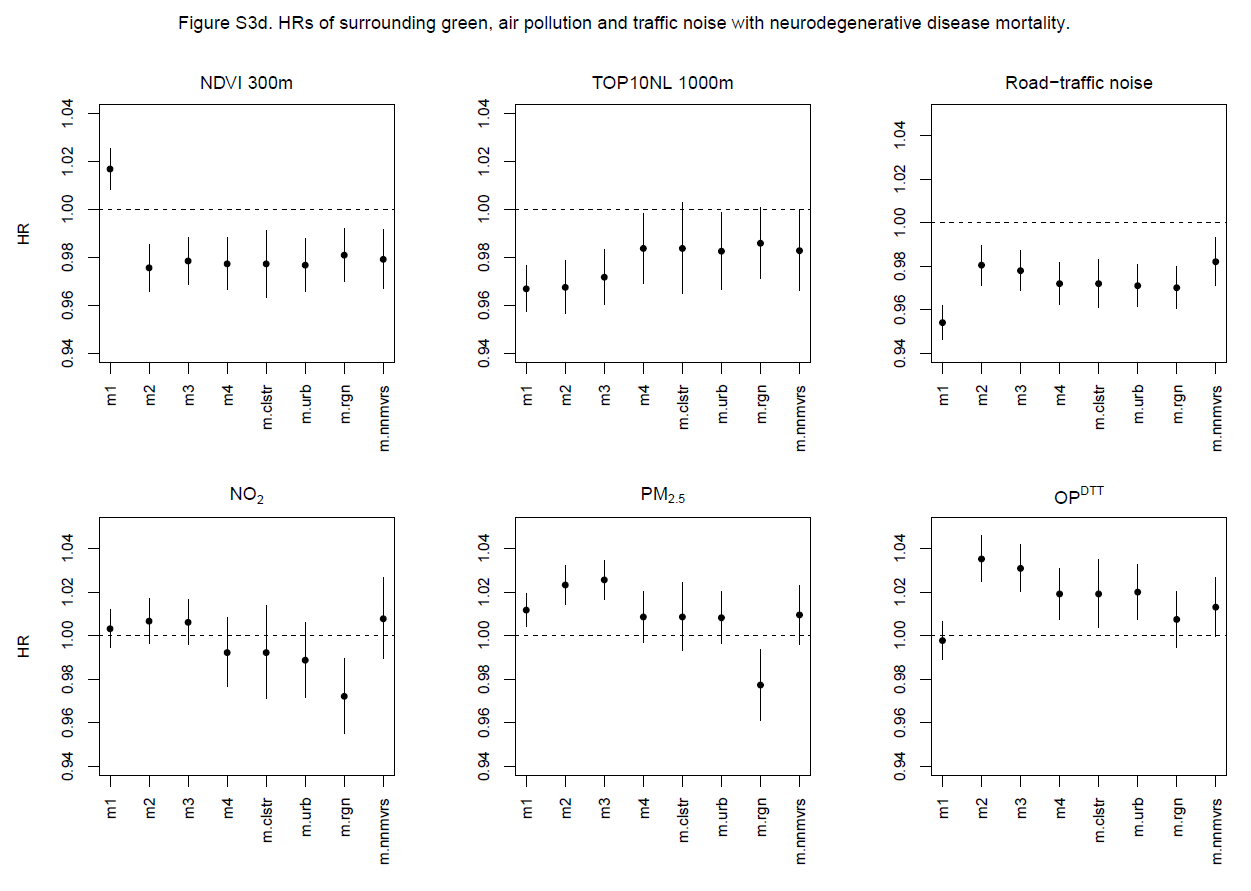


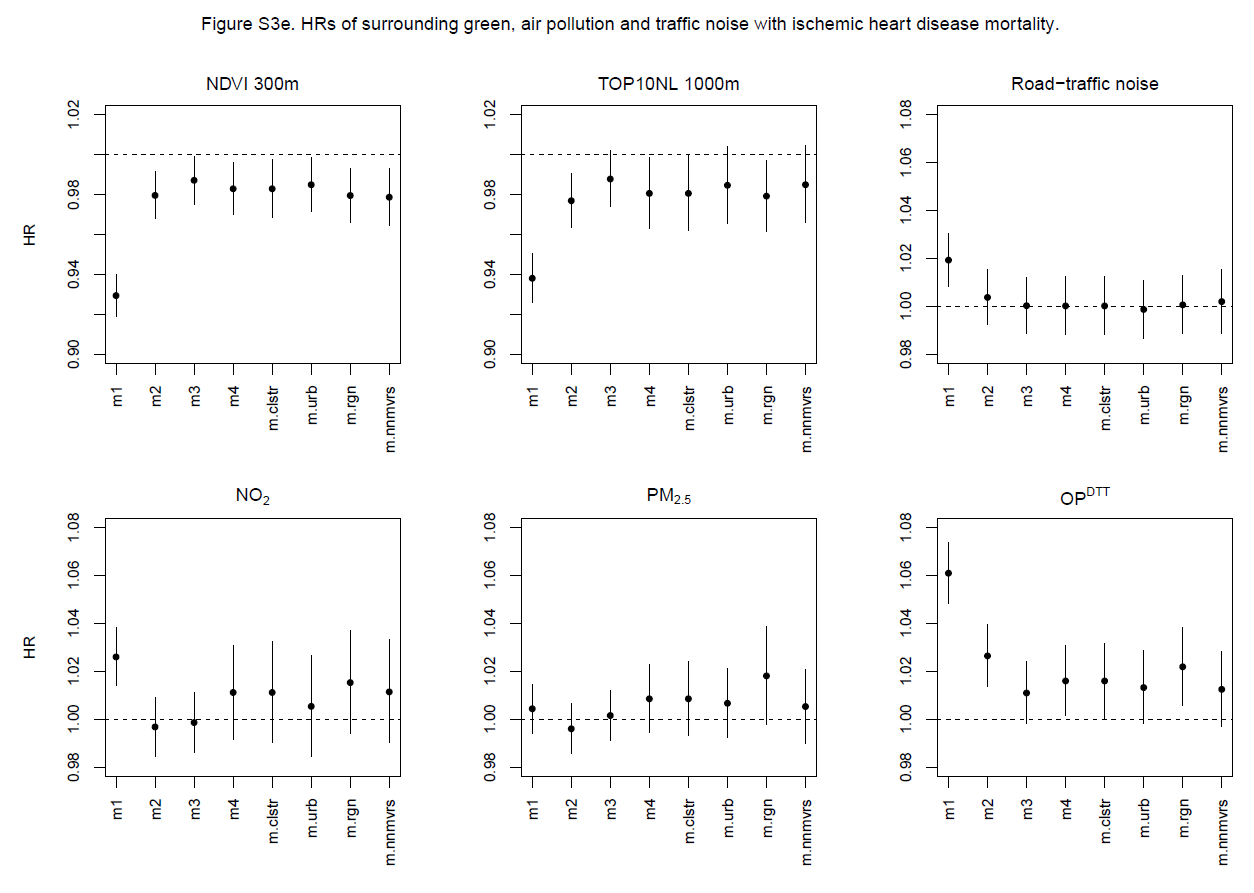


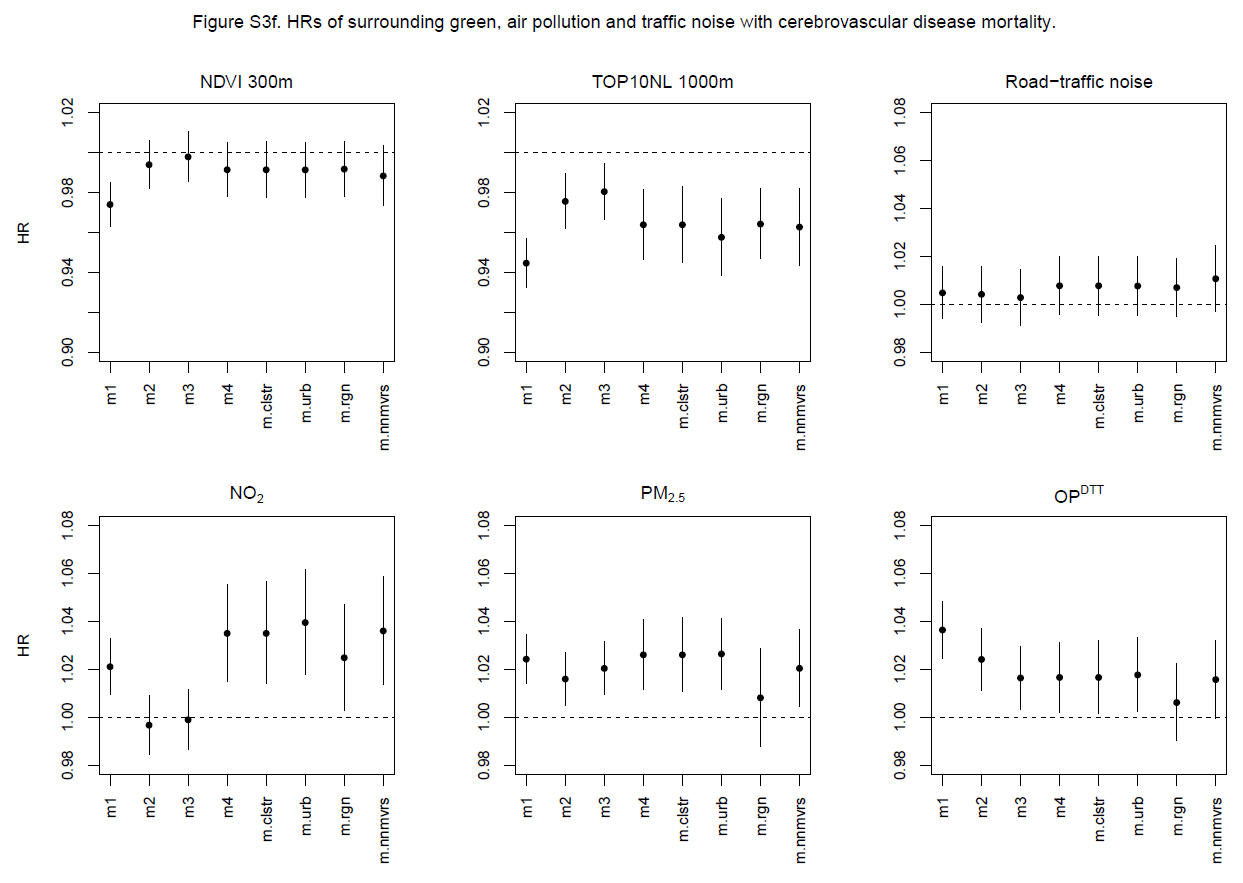


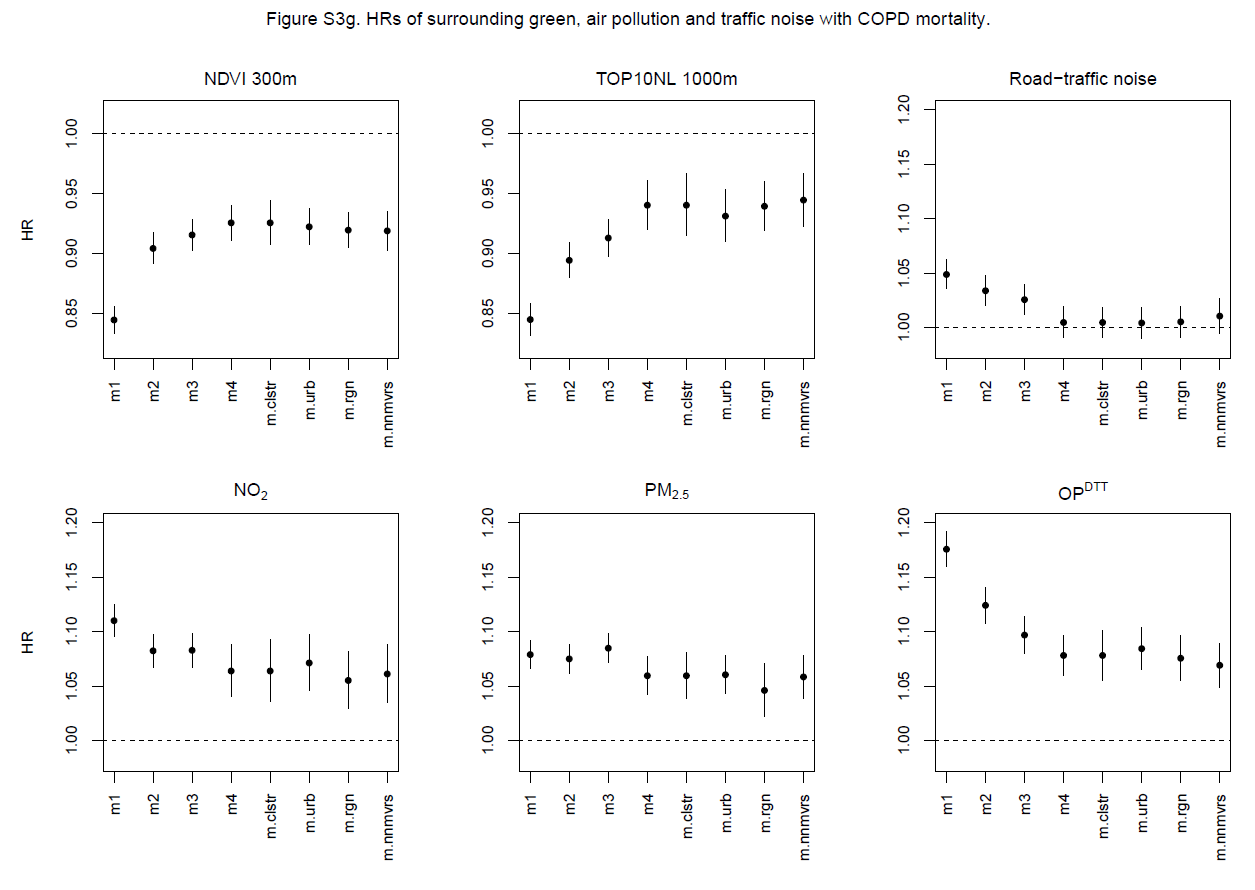


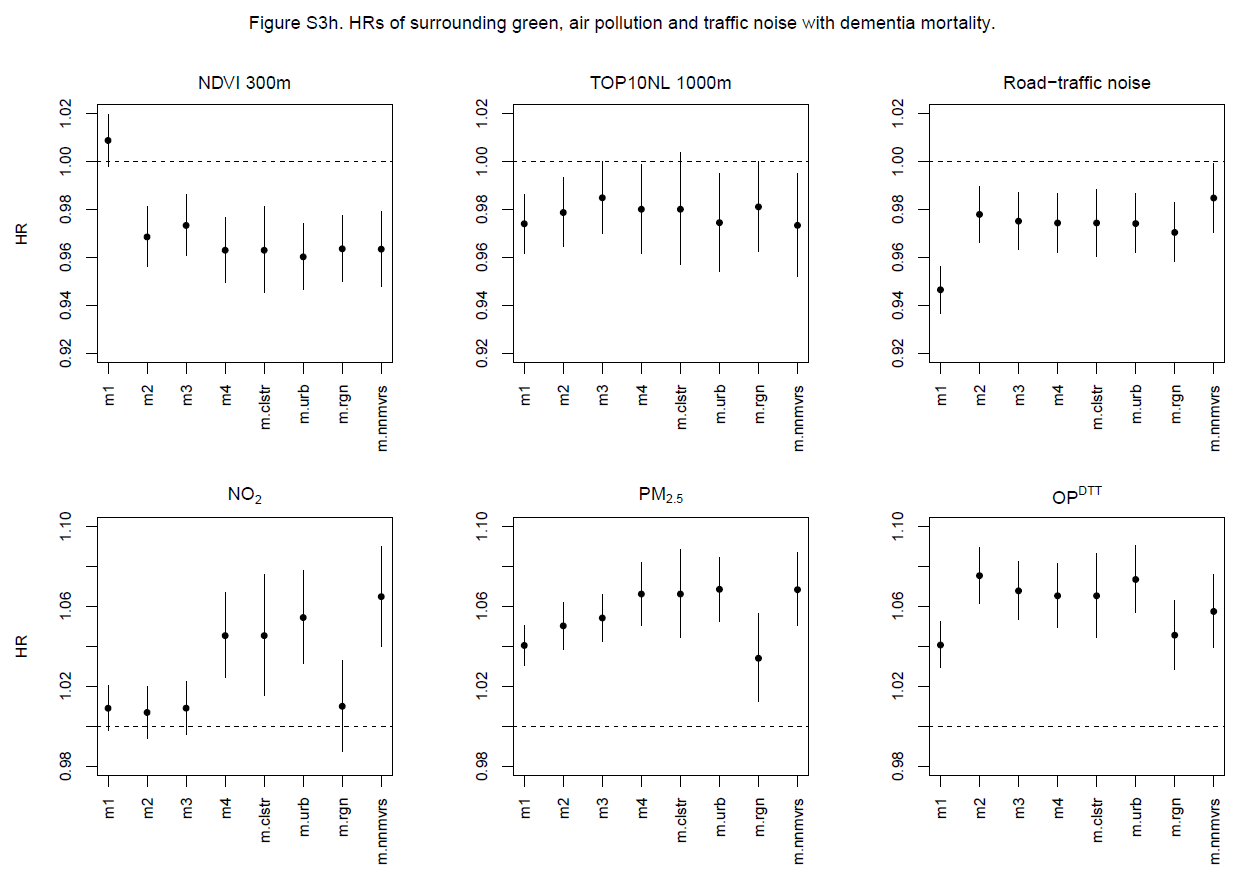


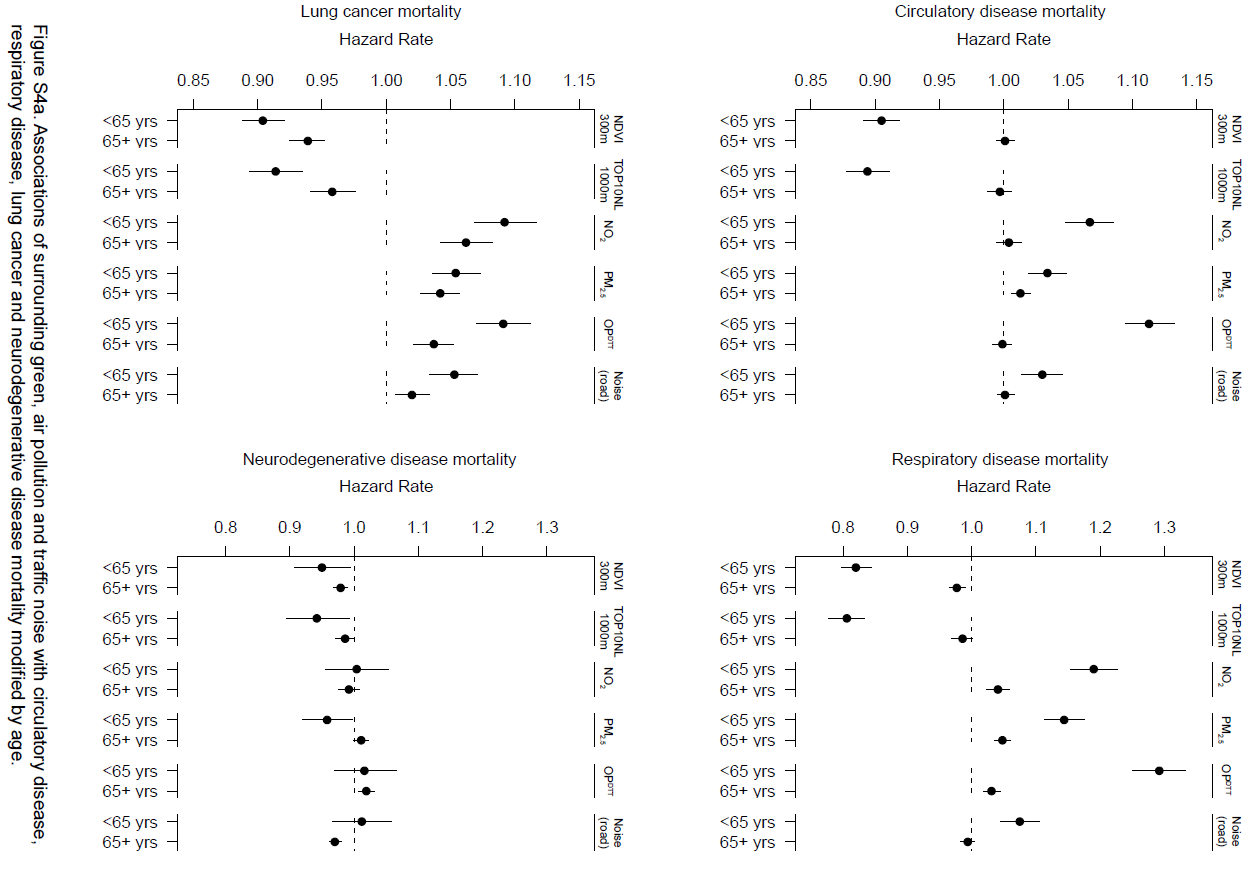


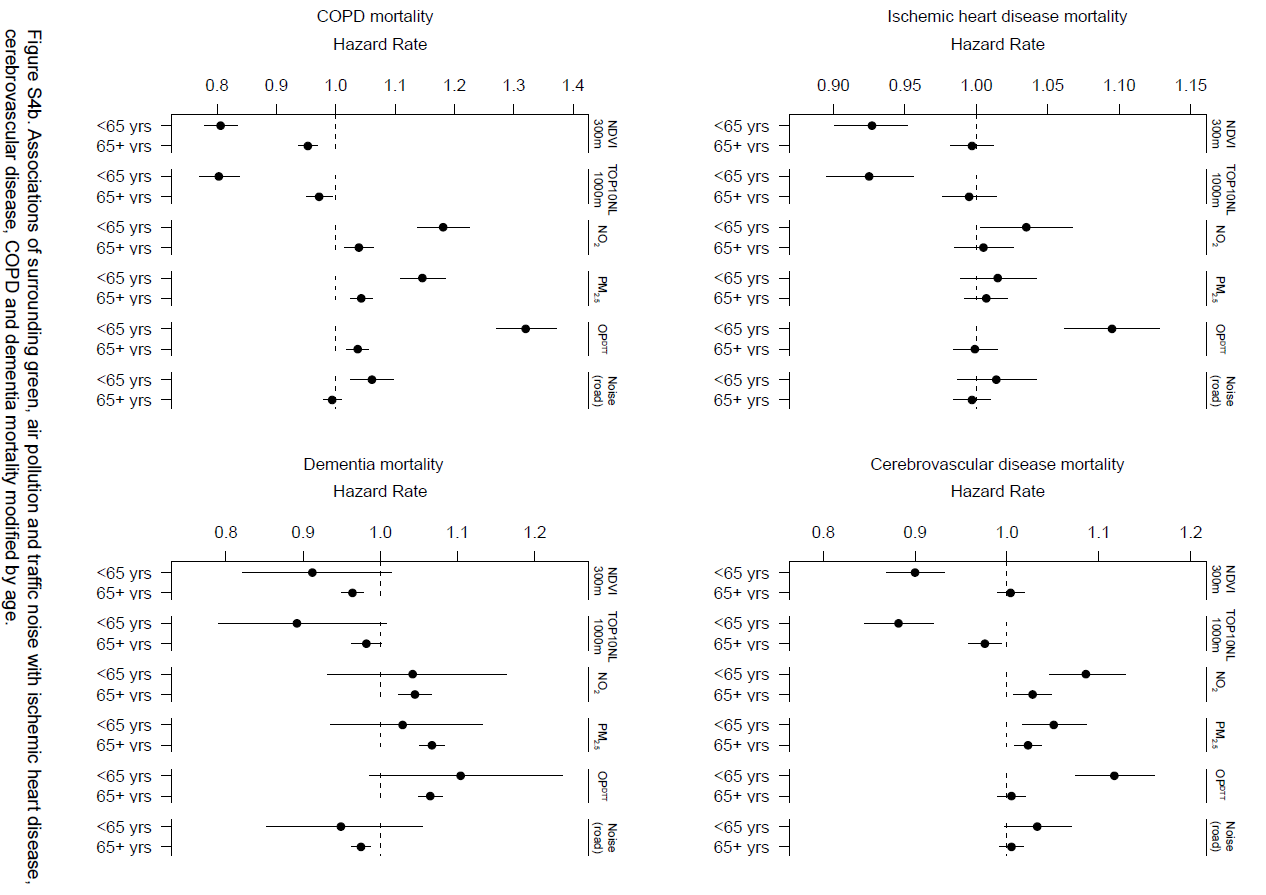


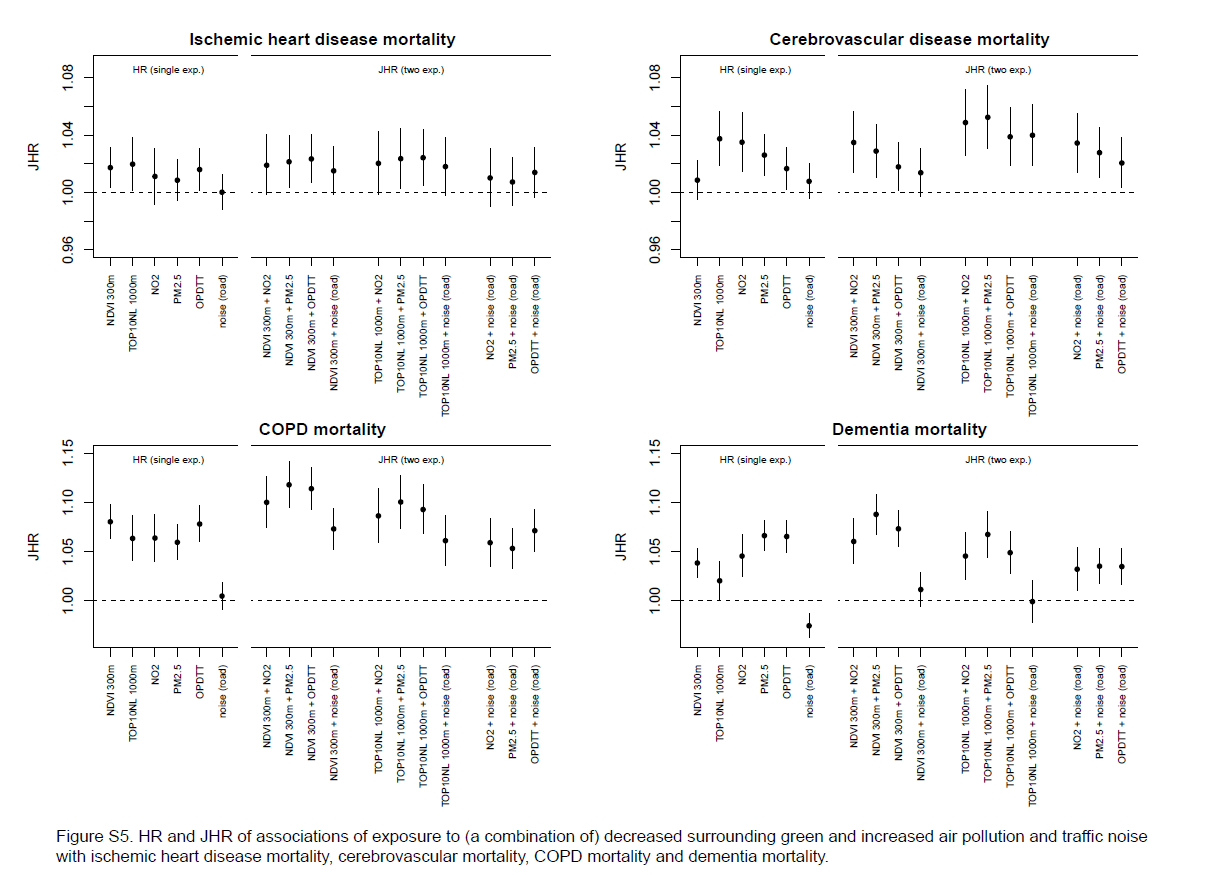

Supplement: Supplementary file 1 — Additional file 1: Table S1. Descriptive statistics of sample of the complete study population and the stratified random sample of the Public health monitor 2012 used in the indirect adjustment method a. Table S2. Associations of NO2, PM2.5 and BC based on the LUR, hybrid and dispersion model with non-accidental, circulatory disease, respiratory disease, lung cancer and neurodegenerative disease mortality in single-exposure models a. Table S3. Associations of exposures with secondary mortality outcomes in single-exposure models a. Table S4. Associations of smoking status and BMI with exposures a, b, c. Table S5. Associations of exposures with secondary mortality outcomes in multi-exposure models a, b. Figure S1. a-i. Estimated exposure−response curves (M4, solid lines) and 95% CIs (dashed lines) for mortality (df=3, density bars are shown on x−axis) a. Figure S2. Spearman rho correlations between surrounding green, air pollution and traffic noise a. Figure S3. a-h. Associations of surrounding green, air pollution and traffic noise with non-accidental mortality in a priori specified models with increasing degree of covariate adjustment and in sensitivity models a. Figure S4. a-b. Associations of surrounding green, air pollution and traffic noise with circulatory disease, respiratory disease, lung cancer, neurodegenerative disease, ischemic heart disease, cerebrovascular disease, COPD and dementia mortality modified by age a, b. Figure S5. HRs and JHRs of associations of exposure to (a combination of) decreased surrounding green and increased air pollution and traffic noise with ischemic heart disease mortality, cerebrovascular disease mortality, COPD mortality and dementia mortality a. [file 12940_2021_769_MOESM1_ESM.docx]
